# Marine Antioxidants from Marine Collagen and Collagen Peptides with Nutraceuticals Applications: A Review

**DOI:** 10.3390/antiox13080919

**Published:** 2024-07-29

**Authors:** Emin Cadar, Ana-Maria Pesterau, Irina Prasacu, Ana-Maria Ionescu, Carolina Pascale, Ana-Maria Laura Dragan, Rodica Sirbu, Cezar Laurentiu Tomescu

**Affiliations:** 1 Faculty of Pharmacy, “Ovidius” University of Constanta, Capitan Aviator Al. Serbanescu Street, No. 6, Campus, Building C, 900470 Constanta, Romania; emin.cadar@365.univ-ovidius.ro; 2 Organizing Institution for Doctoral University Studies of “Carol Davila”, University of Medicine and Pharmacy of Bucharest, Dionisie Lupu Street, No. 37, Sector 2, 020021 Bucharest, Romania; ana-maria.pesterau@drd.umfcd.ro (A.-M.P.); carolina.pascale@drd.umfcd.ro (C.P.); ana-maria-laura.dragan@drd.umfcd.ro (A.-M.L.D.); 3 Faculty of Pharmacy, “Carol Davila” University of Medicine and Pharmacy of Bucharest, Traian Vuia Street, No. 6, Sector 2, 020021 Bucharest, Romania; irina.prasacu@umfcd.ro; 4 Faculty of Medicine, “Ovidius” University of Constanta, University Alley, No. 1, Campus, Building B, 900470 Constanta, Romania; tomescu.cezar.laurentiu@gmail.com; 5 Clinical Hospital C F Constanta, 1 Mai Bvd., No. 3–5, 900123 Constanta, Romania; 6 “Sf. Ap. Andrei” County Clinical Emergency Hospital, Bvd. Tomis, No. 145, 900591 Constanta, Romania

**Keywords:** marine antioxidant, marine nutraceuticals, marine collagen, marine collagen peptides

## Abstract

Collagen peptides and marine collagen are enormous resources currently utilized. This review aims to examine the scientific literature to determine which collagen peptides derived from marine sources and which natural active antioxidants from marine collagen have significant biological effects as health-promoting nutraceuticals. Marine collagen is extracted from both vertebrate and invertebrate marine creatures. For vertebrates, this includes fish skin, bones, scales, fins, and cartilage. For invertebrates, it includes mollusks, echinoderms, crustaceans, and poriferans. The method used involved data analysis to organize information for isolating and identifying marine biocompounds with antioxidant properties. Specifically, amino acids with antioxidant properties were identified, enabling the use of hydrolysates and collagen peptides as natural antioxidant nutraceuticals. The methods of extraction of hydrolyzed collagen and collagen peptides by different treatments are systematized. The structural characteristics of collagen, collagen peptides, and amino acids in fish skin and by-products, as well as in invertebrate organisms (jellyfish, mollusks, and crustaceans), are described. The antioxidant properties of different methods of collagen hydrolysates and collagen peptides are systematized, and the results are comparatively analyzed. Their use as natural antioxidant nutraceuticals expands the range of possibilities for the exploitation of natural resources that have not been widely used until now.

## 1. Introduction

Nutraceuticals have garnered significant attention for their role in alternative treatments for disease prevention and health maintenance. In the European Union (EU), there is specific legislation governing the marketing of functional foods and nutraceuticals, emphasizing their “safety” [1]. The scientific risk assessment is carried out by the European Food Safety Authority [1]. The impact of the COVID-19 pandemic required serious analysis to assess the extent to which dietary supplements and nutraceuticals had potential in the COVID-19 crisis [2,3]. Nutraceuticals are those nutritional products that have additional health benefits [4,5]. Nutraceuticals not only supplement the diet but also contribute to the prophylaxis or treatment of a disorder or disease [6]. Nutraceuticals with antioxidant potential have gained wide interest. In the body, by-products of normal metabolic reactions such as normal cellular respiration and responses to external stimuli on cells generate reactive oxygen species (ROS), which are highly oxidative [7]. Reactive species can be singlet oxygen, hydroxyl radical, superoxide anion, peroxide, and nitrous oxide. Long-term exposure to oxidative stress impairs the biosynthesis of molecules and causes some chondral diseases [8]. Excessive accumulation of ROS damages cell membranes and biological macromolecules, causing damage to tissues and organs, and can generate various pathological conditions such as aging phenomena, arthritis, Alzheimer’s, cancer, and other degenerative diseases [8,9]. To stop such accumulations and maintain the average level of ROS species in the body, antioxidants are needed [10,11]. Synthetic antioxidants including butylated hydroxytoluene (BHT), butylated butylated hydroxyanisole (BHA), and tertiary butylated hydroquinone (TBHQ) are the best known. Although they are compounds with remarkable antioxidant potential, they have shown increased toxicity and their use has begun to be restricted [11]. Under these conditions, natural antioxidants have attracted attention [12]. Compounds with antioxidant capacity from marine resources have gained wide interest, including those from marine fish, seaweed, jellyfish, and mollusks [13,14,15,16]. Among the natural compounds with good antioxidant action and outstanding degradability, the following have stood out: alongside polysaccharides and collagen, gelatin, and collagen peptides [17,18,19]. Native collagen, collagen hydrolysates, and gelatin have gained new potential uses due to their biocompatibility. These include applications as a food source and in various biological and medical domains [20,21,22]. Additionally, they are utilized as biomaterials for medical purposes and in food packaging [23,24]. For a long time, collagen was extracted from terrestrial sources like cattle and pigs. As shown by Lim et al. (2019) due to religious restrictions (Muslims, Hindus, and Jews avoid products from these animals) and the emergence of communicable diseases such as bovine spongiform encephalopathy (BSE), foot-and-mouth disease (FMD), and transmissible spongiform encephalopathy (TSE), which have become prevalent worldwide in recent decades, attempts have been made to search for other sources of collagen [25]. Terrestrial animal products can transmit these diseases (Salvatore et al., 2020) [26]. Thus, collagen from marine resources began to gain great importance. To avoid this risk, Geahchan et al. (2022) and Prelipcean et al. (2022) recommend using marine collagen in wound healing [27,28]. There was an urgent need to identify new alternative sources of collagen. Recent studies on the molecular structure and biochemical properties of fish collagen have shown several similarities to collagen from terrestrial mammals. However, fish collagen has a lower molecular weight and a lower denaturation temperature than mammalian collagen as observed by de Melo Oliveira et al. (2021) and El Blidi et al. (2021) [29,30]. Marine collagen has been studied for applications in different fields: biomaterials, Gallo et al. (2020) and Benayahu et al. (2018); wound healing, Gaspar-Pintilescu et al. (2021) and Cadar et al. (2023); diet use, Paul et al. (2019); and cosmetics, Rodriguez et al. (2018); and antioxidant properties have been reported in several studies by Ballatore et al. (2020), Bashir et al. (2020), Kisling et al. (2019), and Pezeshk et al. (2019) [31,32,33,34,35,36,37,38,39,40]. The marine environment offers a vast resource for isolating collagen and collagen peptides, often wasted as by-products from fish and invertebrate organisms. At present, these resources remain underutilized. In conclusion, the potential of marine-derived collagen antioxidants as valuable nutraceuticals is not fully recognized. This review aims to gather and organize information on techniques for isolating and separating collagen and collagen peptides from marine organisms, both vertebrates and invertebrates, while emphasizing their antioxidant properties. Specifically, it explores the potential of using fish by-products—such as skin, bones, scales, swimming fins, and fish heads—which are rich in collagen and collagen peptides but are currently underutilized globally. The data presented cover the structure and amino acid composition of collagen and their associated antioxidant properties. Results from various analytical methods demonstrate the antioxidant activity of marine collagen products. In addition, the data detail the antioxidant effects of collagen and marine collagen peptides in various medical conditions, supporting their use as natural antioxidant nutraceuticals.

## 2. Data Collection Method

Literature data covering the period 2015–2024 were collected from databases such as Science Direct, SCOPUS, Google Scholar, and Web of Science, where the keywords “marine collagen”, “marine collagen peptides”, or “marine antioxidant” were used for literature data extraction and analysis.

## 3. Isolation of Collagen from Marine Resources

### 3.1. Marine Sources of Collagen

**Figure 1:**
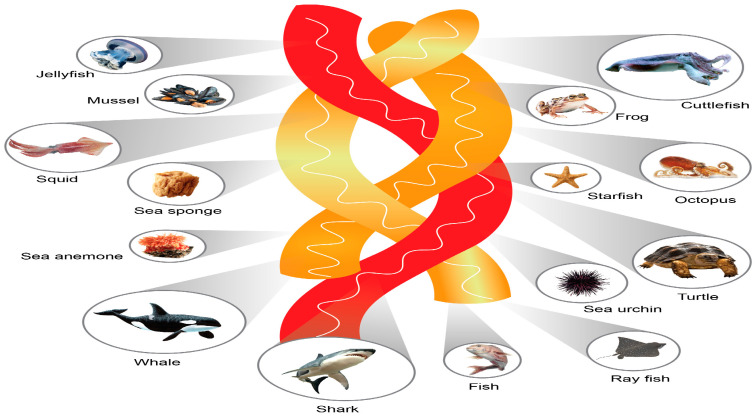
Marine sources for the preparation of marine collagen.

The marine ecosystem encompasses a wide range of habitats, from the surface waters to the deep sea, which host a diverse array of species. These species are a valuable ecological source for obtaining marine collagen with antioxidant properties. The raw materials for marine collagen can be extracted from both vertebrates and invertebrates, including marine fish (such as fish skin and fish waste), poriferans (marine sponges), mollusks (byssus and cephalopods), crustaceans (mantis shrimps), echinoderms (starfish and sea urchins), and coelenterates (jellyfish). Figure 1 shows marine organisms that may be potential sources of marine collagen.

The diversity and potential were highlighted by de Melo Oliveira et al. (2021) and Rahman (2019) [29,41]. Marine collagen can vary significantly in structure, depending on its source. It is worth noting that marine vertebrates, such as marine fish, possess more intricate skeletal systems with abundant collagen in their bones and skin. This observation is supported by Cherim et al. (2019) and Prajaputra et al. (2024) [42,43]. Currently, a major problem in the fish farming industry is the inadequate management of waste or by-products resulting from improper fish processing, which leads to economic losses and environmental problems.

### 3.2. Marine Collagen Extraction

#### 3.2.1. Extraction Procedures

Biotechnologies used to extract collagen from marine organisms have been detailed in studies by Prajaputra et al. (2024) and Jafari et al. (2020), who categorized them based on the extracted collagen type. These methods include alkali-soluble collagen (SSC), acid-soluble collagen (ASC), enzymatic methods (PSC), and ultrasonic methods [43,44]. Additionally, Cherim et al. (2017) and Lu et al. (2023) have reported on the isolation and characterization of collagen from marine sources [45,46]. Depending on the chosen extraction method, collagen products vary in different yields and properties. Marine collagen extraction typically involves two primary steps:

I. The pre-treatment stage involves preparing the raw material and eliminating contaminants to ensure the purity of the final product. Marine by-products, including skin, bones, scales, or the head and appendages of marine organisms in the case of invertebrates, are carefully selected. Various compounds, such as pigments, non-collagenous proteins, and unwanted lipids, are removed during this stage, as documented by Ampitiya et al. (2023) [47]. Additionally, other researchers, such as Wang et al. (2018) and Chen et al. (2021) have reported successful removal of adhesive residues using aqueous NaCl solutions of varying concentrations [48,49]. Cumming et al. (2019) reported the removal of inorganic minerals by demineralization with EDTA (ethy-lenediaminetetraacetic acid), as reported [50]. Another option was the use of a 0.5 M HCl solution, which was reported by Xu et al. (2017), Kıyak et al. (2024), and Li C. et al. (2020) [51,52,53]. Sodium chloride, n-butanol, and sodium hypochlorite hexane or hydrogen peroxide solution were used for the removal of dyes and non-collagenous fats, as reported by Wang et al. (2018), and Liu et al. (2019) [48,54]. In 2021, Song et al. reported that fermentation is also an alternative pretreatment that helps to obtain collagen from Nile tilapia skin by the PSC process with very good purity [55].
II. The actual extraction stage can be carried out by specific methods. The most commonly used methods for obtaining collagen are the ASC and PSC methods [43,56].

The ASC procedure is the most widespread. Sirbu et al. in 2019 reported studies on the validation of a quantitative method for the extraction of collagen from the skin of gray mullet fish by the ASC process [57]. For the extraction of collagen from the tissues of marine organisms, acetic acid is the most widely used dilute acid, but other acids can also be used, such as citric acid, lactic acid, or chloroacetic acid. In 2020, Senadheera et al. and in 2021, Shaik et al. showed that organic acids provide higher collagen extraction efficiency than inorganic acids [58,59]. The most widely used ASC extraction method is the one using acetic acid in a 0.5 M concentration, continuously stirred between 24 h and 72 h, for collagen extraction [43,56]. From multiple reported studies, it appears that in order to obtain the best extraction results, the acetic acid concentrations must be adapted to the sample type. Thus, Hadfi et al. (2019) extracted collagen from silver catfish (*Pangasius* sp.) skin with different concentrations of acetic acid (0.5 M and 0.7 M) and reported yields of 10.9% and 5.47%, respectively [60]. So, there was a higher yield when 0.5 M acetic acid concentrations were used [60]. However, Baderi N.A. et al. (2019) extracted collagen from shortfin scad (*Decapterus macrosoma*) and reported 1.01% and 1.31% yields when using 0.5 M and 0.7 M acetic acid, respectively, so the yield was higher at 0.7 M acetic acid concentrations [61]. In the following step, the collagen supernatant is obtained by centrifugation, which then has to be precipitated with salt (NaCl). This separates the collagen precipitate. In 2020, Seixas et al. reported these methods along with other procedures for the extraction of collagen from elasmobranch by-products for potential biomaterial use [62]. In 2018, Tanaka et al. isolated collagen from bluefin tuna (*Thunnus orientalis*) skin, and Tan et al. isolated collagen from channel catfish (*Ictalurus punctatus*) skin [63,64].

The PSC procedure is also a commonly used process and is based on the reaction of collagen with pepsin. Venkatesan et al. (2017), showed that in this treatment, the enzymes provide increased yields and purity of collagen [65]. Zhao et al. (2018) showed that acid-soluble collagen tends to generate a lower yield, and pepsin extraction increases extraction yield because pepsin cleaves crosslinks in the telopeptide region, thus producing increased collagen solubility in acid [66]. Castaneda-Valbuena et al. (2022) found that treating certain proteins with pepsin reduces their allergenicity, making this treatment suitable for producing collagen hydrolysates or peptides [67]. To obtain collagen hydrolysates, the collagen macromolecules need to be broken down further through processes like basic, acidic, or enzymatic hydrolysis [67]. Asaduzzaman et al. (2020) demonstrated that acidic or basic conditions, along with subcritical water hydrolysis (which avoids toxic solvents and collagen degradation), are preferable for collagen degradation [68]. Pepsin treatments for collagen extraction have been reported by Asaduzzaman et al. (2020) for collagen from mackerel bones (*Scomber japonicus*) and skin, as well as by Zhang et al. (2017) for frog skin (*Rana nigromaculata*) using a 0.5 M acetic acid extract containing 0.1% pepsin for 72 h [68,69].

#### 3.2.2. Procedures Applied to the Isolation of Collagen from Invertebrates

In the case of other invertebrate marine organisms, it has been necessary to resort to adapted procedures for collagen extraction. For example, jellyfish collagen is generally precipitated with an aqueous solution of 2.3–2.6 M NaCl.

The collagen precipitate is collected, centrifuged, and solubilized in a 0.5 M acetic acid solution (about three days), followed by salting by dialysis with a NaHPO4 solution. The precipitated collagen is separated by centrifugation, then solubilized in acetic acid and purified by reprecipitation with the addition of solid NaCl to a concentration of 0.9 M. Acid-soluble collagen (ASC) can be digested with pepsin to obtain atelocollagen [19]. In the case of sea urchins, the intact collagen fibrils in the peristomal membranes are different from other types of collagen and cannot be extracted by traditional acid solubilization methods, as this method generally produces it as hydrolyzed gelatin. The shredded native tissue is sequentially treated with a hypotonic solution and a specific decellularization solution to remove both cellular debris and skeletal parts and pigments. After 3–4 days in the β-mercaptoethanol disaggregating solution, collagen fibers are obtained, which are then passed through a filtration step and dialyzed in a 0.5 M EDTA-Na solution [19]. The same protocol is employed for extracting collagen fibers from the aboral arm walls of the starfish. However, an additional step is introduced wherein the samples undergo treatment with 1 mM citric acid between the decellularization and disaggregation solutions. This step is crucial for eliminating calcium carbonate osmosis present in the fresh tissue [19]. In a study conducted by Sun et al. (2021), soluble collagen (ASC), pepsin-soluble collagen (PSC), and water-soluble gelatin (WSG) were extracted from squid (*Dosidicus gigas*) skin. They found that using the ASC process at 4 °C resulted in the lowest yield of 33.5% [70]. The addition of pepsin (PSC process) increased the collagen yield by approximately 35.0%. The highest yield of 81.9% was achieved through water extraction at 60 °C (WSG). The authors demonstrated that low temperatures can effectively preserve the native helix structures of ASC and PSC. In contrast, heat treatment led to the transformation of collagen into gelatin with uncoordinated and denatured structures [70]. Antioxidant peptides derived from marine fish are obtained by enzymatic hydrolysis methods using different types of enzymes (alkalase, α-chymotrypsin, neutrase, papain, pepsin, and trypsin). Castaneda-Valbuena et al. (2022) showed that the use of optimized buffer systems is required for these enzymes [67]. Separation of peptides is carried out by using chromatographic techniques and ultrafiltration membranes. After collecting the peptide fractions, the lyophilization step follows to obtain purified peptides [67].

**Figure 2:**
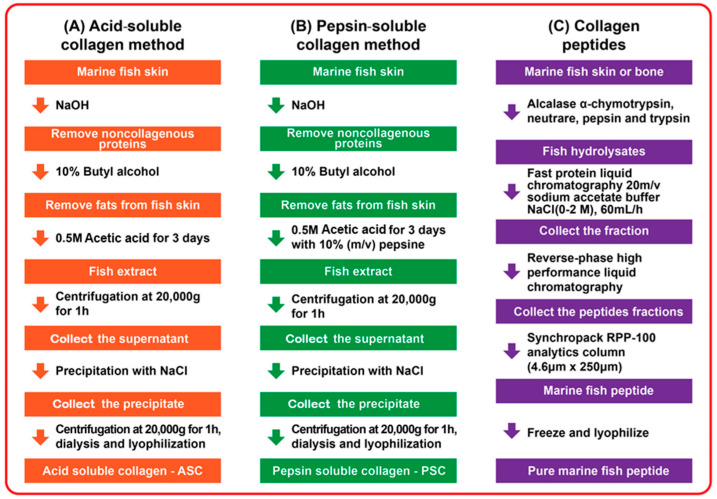
Scheme for obtaining marine collagen through (**A**) acid-soluble collagen method; (**B**) pepsin-soluble collagen method and (**C**) collagen peptides.

Figure 2 illustrates the commonly employed methods for extracting marine collagen from fish. These include the following: (A) acid treatment, (B) enzymatic treatment, and (C) extraction using pepsins for marine collagen [65]. Additionally, Figure 2 outlines the general procedures for generating collagen peptides from fish skin and bones [65].

#### 3.2.3. Ultrasonic Procedure

The ultrasonic protein extraction process is simple, fast, risk-free, reliable, and financially beneficial. Ultrasonication leads to increased enzyme activity and helps remove temperature-sensitive chemicals. Shaik et al. (2021) studied the effect of ultrasound on collagen extraction in ASC and PSC procedures and showed that the method, being non-invasive, can obtain collagen with an almost intact structure [59]. However, prolonged exposure to ultrasound can lead to a cavitational effect, resulting in elevated temperatures, shear forces, and pressures within the medium. This effect causes the disruption of hydrogen bonds and van der Waals interactions in polypeptide chains, ultimately leading to protein denaturation. Despite these drawbacks, studies such as Shaik et al.’s (2021) have observed the application of ultrasound-assisted ASC and PSC treatments, demonstrating increased yields for collagen extracted from *Sharpnose stingray* (*Dasyatis zugei*) using both acid extraction and ultrasound-assisted pepsin extraction while preserving other properties [59]. Zou et al. (2017), Ali et al. (2018), and Petcharat et al. (2021) showed that ultrasound treatment at 20–35 kHz, amplitude 20–100%, pulse 2/2 s–20/20 s, and 200–750 W lasts about 10–30 min or even 0–24 h [71,72,73]. Ali et al. (2018) found that golden carp (*Probarbus jullieni*) skin extracted with pepsin followed by ultrasonication produced a higher content of amino acids and an increased denaturation temperature, so the combined extraction method maintained the triple helical structure of extracted collagen [72]. Petcharat et al. (2021) performed collagen extraction on clown featherback (*Chitala ornata*) skin using ultrasonic methods [73]. Pezeshk et al. (2022) confirmed by modern physicochemical methods (X–ray diffraction and FTIR) that collagen from yellowfin tuna skin extracted with ultrasound showed a native undenatured triple catenary helical structure, so ultrasonication did not affect the structural integrity of the collagen [74]. In conclusion, the application of ultrasound in collagen extraction reduces extraction time and can increase both the quality and quantity of extracted collagen at certain extraction amplitudes and times.

#### 3.2.4. Other Methods

**Figure 3:**
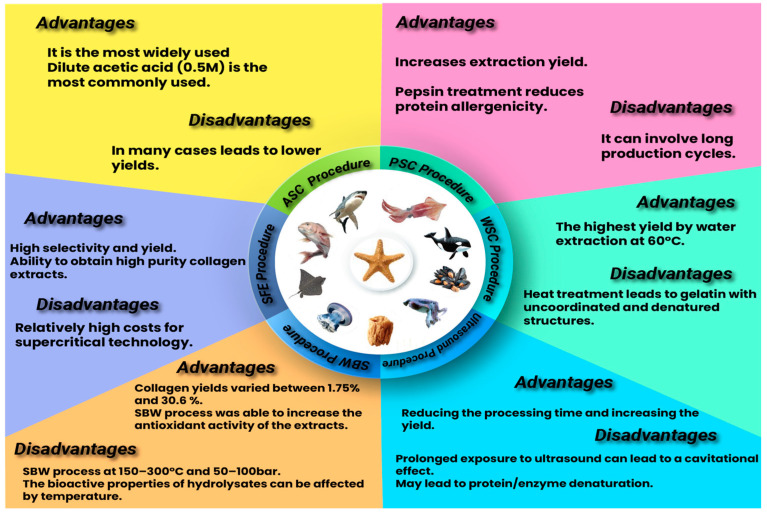
Advantages and disadvantages of marine collagen extraction procedures.

There are alternative methods for extracting collagen from marine resources; however, they are not as popular as ASC, PSC, and ultrasonic treatments [52]. Figure 3 shows the marine collagen extraction procedures with their advantages and disadvantages.

The WSC procedure has been used to extract collagen from marine invertebrates [70]. This water-soluble collagen (WSC) is produced at 60 °C and is relatively easy to make. However, the process ultimately transforms the collagen into gelatin with uncoordinated and denatured structures, as demonstrated by Sun et al. (2021) [70].

The subcritical water hydrolysis (SBW) procedure represents a green alternative to traditional methods. It involves using water at temperatures between 150 and 300 °C and pressures between 50 and 100 bar. Kıyak et al. (2024) demonstrated that this method has been successfully used for extracting collagen from various fish species and fish by-products [52]. However, a disadvantage of SBW is that the high temperatures may affect the collagen structure [52].

The supercritical fluid extraction (SFE) procedure is an alternative to traditional extraction methods. SFE uses a supercritical fluid, typically CO_2_, as the extracting solvent to separate components. CO_2_ is preferred due to its numerous advantages. The primary benefit of SFE is the ability to obtain purified components. Figure 3, as presented by Kıyak et al. (2024), outlines additional advantages and disadvantages of the SFE method [52].

### 3.3. Data on the Isolation of Marine Collagen

#### 3.3.1. Marine Collagen Isolated from Leather and Marine Fish Waste

Marine fish belong to the vertebrate category, and the raw materials used to isolate collagen from fish are skin, bones, scales, cartilage, and other by-products (such as swimming fins). Fish by-products can vary in composition depending on the size of the fish, the species, and the technology used to process them. Type I collagen obtained from these by-products is preferred. Among the research carried out for the extraction of collagen from skin fish, we list the isolation collagen from *Alu*—*Alu* (*Sphyraena* sp.) by Matarsim et al. (2023) [75]. The extraction of collagen from skins of Asian sea bass and Spanish mackerel (*Scomberomorus commerson*) was performed by Ampitiya et al. (2023) [47]. Collagen and collagen peptide excision from the skin of round goby fish (*Neogobius melanostomus*) by Yemisken et al. (2023) and from the skin of silver catfish (*Pangasius* sp.) by Shaik et al. (2023) have been reported [76,77]. Type I collagen was extracted from other fish by-products, such as unicornfish (*Naso reticulatus*) bones obtained by Fatiroi et al. (2023) [78]. Research has been reported to isolate collagen from parrotfish (*Scarus sordidus*) scales by Jaziri et al. (2023) and from Megalonibea (*Megalonibea fusca*) swim bladders obtained by Mo et al. (2023) [79,80]. Marine collagens were also obtained from the swim bladder of Totoaba (*Totoaba macdonaldi*) extracted by Cruz-Lopez et al. (2023), from the swim bladder of sea eels (*Muraenesox cinereus*) extracted by Li H. et al. (2023), and from the cartilage of blue sharks (*Prionace glauca*) by Pan et al. (2023) [81,82,83]. Research on the extraction of marine collagen from different fish by-products was reported, including from the bones of lizardfish (*Saurida tumbil*) by Jaziri et al. (2022), and from the tail tendon of skipjack tuna (*Katsuwonus pelamis*) by Chanmangkang et al. (2022) [84,85]. Marine collagen was isolated from the swim bladder of grass carp (*Ctenopharyngodon idella*) by Dong et al. (2022), and from the skin of Greenland halibut (*Reinhardtius hippoglossoides*) by Martins et al. (2022) [86,87]. Other research to obtain marine collagen was done from catfish (*Silurus triostegus*) skin by Abbas et al. (2022) and from dusky grouper (*Epinephelus marginatus*) scales by Tziveleka et al. (2022) [88,89]. Collagen was isolated from shark (*Prionace glauca*) cartilage by Seixas et al. (2020) and from surgeon fish (*Huso huso*) skin by Atef et al. (2020) [62,90]. Zhang et al. (2022) reported data on gelatin from the cartilage of Siberian sturgeons (*Acipenser baerii*) [91]. Type I collagen was extracted from the swim bladder of giant croakers (*Nibea japonica*) by Chen et al. (2019) and from the skin of bigeye tuna (*Thunnus obesus*) by Ahmed et al. (2019) [92,93]. Kittiphattanabawon et al. (2019) also extracted collagen from Nile tilapia (*Oreochromis Niloticus*) scales by ASC and PSC procedures [94]. Studies for the extraction of marine collagen from the skin of silver catfish (*Chrysichthys nigrodigitatus*) were reported by Hukmi et al. (2018) [95]. Iskandar et al. (2018) extracted collagen from the skin of bonylip barb fish (*Osteochilus vittatus*) [96]. Changfeng C. et al. (2013) characterized collagens from the cartilage of the Scottish hammerhead (*Sphyrna lewini*), and Zhong-Rui reported data on collagens from the skin and bone of the Spanish mackerel (*Scomberomorous niphonius*), [97,98]. Hu et al. (2023) reported data on the utilization of peptides from the collagens of monkfish (*Lophius litulon*) swim bladders [99]. Li et al. (2018) reported studies obtaining collagen from scales of the Miiuy croaker (*Miichthys miiuy*) [100]. Other studies on the isolation and valorization of collagen from fish and fish derivatives were reported. Nurmila et al. conducted research on the extraction and characterization of antioxidant activities from yellowfin tuna *Thunnus albacares* skin [101,102]. Studies concerning collagen from skin of *grey mullets* from the Black Sea were also reported by Cherim et al. in 2019 and in 2017 [103,104]. Collagen extracted from the skin of bluefin tuna (*Thunnus orientalis*) was reported by Tanaka et al. (2018) [63].

#### 3.3.2. Collagen from Marine Invertebrates

Collagen isolation from invertebrates has been relatively less studied. Sea sponges, sponges or poriferans are part of a category of invertebrates that have been shown to be a potential source of collagen, although they have been little investigated. To date, about 8500 species are known. The class *Demospogiae* includes *Chondrosia reniformis*, which has been studied as a potential collagen source by Tassara et al. (2023), Araújo et al. (2021), and Pozzolini et al. (2018) [105,106,107]. Fernandes et al. (2021) reported studies on the biological performance of marine sponge collagen [108]. Parisi et al. (2019) reported on the biological activities of materials derived from spongin, a form of collagen from marine sponges, when incorporated into other materials [109].

Langasco et al. (2017) explored the use and enhancement of the natural collagen-horny skeleton of marine sponges (*Porifera*, *Dictyoceratida*) as a biologically based dressing for topical drug delivery [110].

Table 1 shows recent studies with data on the part of the body analyzed, the type of extraction method, the yield obtained for collagen, data on collagen analysis methods for identification, and the type of collagen identified.

In addition to marine sponges, echinoderms of the *phylum Echinodermata*, which includes five distinct classes, were also studied for their collagen. Vate et al. (2023) investigated collagen in the common starfish (*Asterias rubens*), while Han et al. (2021) studied collagen in the starfish (*Asterias pectinifera*) [111,112]. Li et al. (2020) extracted a high percentage of collagen, up to 72%, from the sea cucumber *Holothuria cinerascens*, demonstrating its potential as a marine collagen resource [113]. Tian et al. (2020) also extracted collagen from the sea cucumber *Apostichopus japonicus* [114]. Another promising source of marine collagen is the *Coelenterates*. Esparza-Espinoza et al.’s (2019) remarkable research involved extracting collagen from the jellyfish *Stomolophus meleagris* [115]. Additional studies include those conducted by Felician et al. (2019), who extracted collagen from *Rhopilema esculentum*, and Rastian et al. (2018), who worked with *Catostylus mosaicus jellyfish* [116,117]. Khong et al. (2018) isolated collagen from the jellyfish *Acromitus hardenbergi*, and Cheng (2017) focused on *Rhopilema esculentum* [118,119]. CunhaNeves et al. (2022) reported studies on blue mussel (*Mytilus edulis*) byssus collagen hydrolysates, and Rodríguez, F et al. (2017) reported studies on collagen extraction from mussel byssus [120,121].

Hiransuchalert et al. (2021) extracted collagen type I from different mantis shrimp species [122]. Wu et al. (2019) reported studies on collagen isolated from *Coelomactra antiquate* [123]. Ezquerra-Brauer et al. (2018) reported studies on collagen in jumbo squid (*Dosidicus gigas*) [124]. The high collagen percentages reported in various studies from Table 1 are as follows: 84.81% (PSC) from the swim bladders of *Megalonibea fusca* by Mo et al. (2023), 93.7% (PSC) from the sea eel (*Muraenesox cinereus*) by Li, H. et al. (2023), and 72.2% (PSC) from the sea cucumber (*Holothuria cinerascens*) by Li, P.H. (2020) [80,82,113].

## 4. Marine Collagen Structure and Composition

### 4.1. Structural Characteristics of Collagen and Collagen Peptides

Collagen is a protein found in all living things. This protein has a complex structure consisting of 29 collagen types, as explained by Cherim et al. (2019) and Meyer et al. (2019) [42,125]. In vertebrates, type I collagen is the most abundant type in the body and can be found in bones, skin, tendons, and organs, as explained by Meyer et al. (2019) [125]. Type II collagen is found in cartilage. Type III collagen is present in reticular fibers as well as in blood and skin [125]. In invertebrates, type I and IV collagens are found. By partial denaturation of native collagen, gelatin is obtained, which is a major source of protein biopolymers. Collagen peptides are fragments of collagen with lower molecular masses that are detached from the large triple helix chain. Ryu et al. (2021) showed that proteolytic enzymes can break down proteins into hydrolysates comprising small peptides consisting of 2–20 amino acids [126].

**Figure 4:**
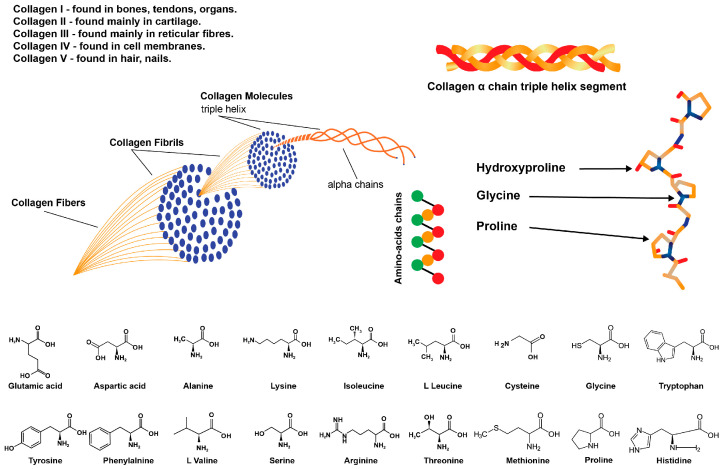
Structure of collagen fibers, collagen fibrils, and amino acid chains. Reprinted with permission from reference [34], 2023, Emin Cadar.

The molecular weight, length, and sequence of peptides, as well as their amino acid composition, influence their bioactive properties; hydrolysates produce amino acid forms that are useful in supporting various human biological functions, as stated by Yathisha et al. (2018) [127]. Zhang et al. (2023) showed the typical collagen structure of fish skin [128]. Al-Shaer et al. (2021) showed that the collagen chain of fish exhibits a *Gly-X-Y* repeat sequence, where X and Y are generally *Pro* and *Hyp*, respectively [129]. Zhu et al. (2020) reported data on type II collagen from the cartilages of skates and sturgeons [130]. Romijn et al. (2019) analyzed the differences between collagen types I and II, and Hu et al. (2022) analyzed the differences generated by the structure of three commercial tuna species with modern methods of analysis [131,132]. Hernández-Ruiz et al. (2023) analyzed the structure of collagen peptide fractions from tilapia (*Oreochromis aureus Steindachner*, 1864) scales [133]. Figure 4 shows the structure of collagen, collagen peptides, and amino acid chains [34]. Also highlighted are the top five collagen types and the locations where they are most abundant.

### 4.2. Amino Acids in Marine Collagen

In vertebrates, different types of collagen show tropocollagen structures. These molecules consist of approximately 35% glycine (*Gly*), 21% proline (*Pro*), 11% alanine (*Ala*), and hydroxyproline (*Hyp*) [126]. Hydroxyproline at the Y-position is believed to enhance the stability of the helical structure. From a nutritional perspective, amino acids are categorized as essential (EAA), non-essential (NEAA), or conditionally essential (CEAA). The concept of functional amino acids (FAA) has also been introduced; these amino acids are involved in and regulate metabolic pathways that improve health, growth, development, survival, reproduction, neurological metabolic diseases, and infectious diseases [126].

**Figure 5:**
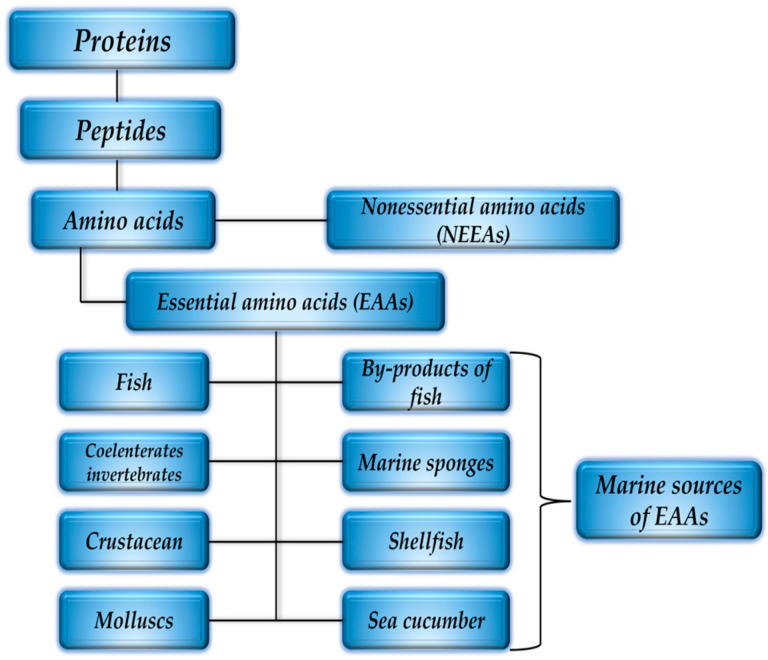
Amino acids (EAA) in marine-derived collagen and collagen peptides.

*Arg*, *His*, *Cys*, *Lys*, *Leu*, *Thr*, *Met*, *Trp*, *Tyr*, and *Val* are EAA; *Pro*, *Glu*, *Gln*, and *Gly* are CEAA; and *Ala*, *Ser*, and *Asp* are NEAA for human nutrition. In human nutrition, *Arg*, *Cys*, *Leu*, *Met*, *Trp*, *Tyr*, *Asp*, *Glu*, *Gly*, and *Pro* have been classified as FAA, as shown by Šimat et al. (2020) [7]. Figure 5 shows the potential marine sources of essential amino acids (EAA).

#### 4.2.1. Amino Acids from Fish Collagen

The amino acid content of collagen in fish is very different depending on the species of fish, the marine habitat in which it lives, and the pollutants present in marine waters, especially in coastal waters. Research reported on the amino acid content of marine collagen extracted from fish skin and fish by-products shows a different distribution of amino acid types. Blanco et al. (2017) determined the amino acid compositions of collagen from *Thunnus albacares* fish and found that this skin residue is rich in *Gly*, *Pro*, *Ala*, and *Glu* [134]. Je et al. (2019) stabilized the amino acid composition of *Tilapia* fish collagen hydrolysates and found the highest values for *Gly*, *Ala*, *Pro*, and *Glu* [135]. Garehgheshlagh et al. (2020) studied the *Rutilus frisii kutum* species and determined that it contained the highest amounts of total amino acids in *Gly*, *Pro*, *Glu*, and *Ala* [136].

Thuy et al. (2020) reported the highest amounts of the total amino acids found in *Gly*, *Pro*, *Ala*, and *Hyp* in *Pangasianodon hypophthalmu*, and for the species *Oreochromis niloticus*, they reported the order of amino acids in *Gly*, *Pro*, and *Hyp* [137]. Truong et al. (2021) reported the study of amino acids in the species *Channa striata* and established the following order: *Gly*, *Hyp*, *Ala*, and *Glu* [138]. Son et al. (2022) reported the amino acid order *Gly*, *Ala*, *Pro*, *Arg*, and *Glu* for both the species *Pagrus major* and *Paralichthys olivaceus* [139]. Rýglova et al. (2023) provided studies on amino acids from skin collagen of the fish *Cyprinus carpio* and stabilized the values in the order *Gly*, *Ala*, and *Pro* [140]. Cruz-Lopez et al. (2023) reported the amino acid composition of collagen extracted from the fish *Totoaba macdonaldi*, with the highest values for *Gly*, *Ala*, *Pro*, and *Glu* [81]. From the presented analysis, we can see that the main amino acids in most of the collagens in pest skin are *Gly*, *Pro*, *Ala*, *Glu*, *Hyp*, and *Val*. The amino acid *Ala*, although belonging to the category of non-essential amino acids, is quantitatively found in all collagen extracts from the skin of the marine fish studied. *Pro* and *Ala* were the most abundant hydrophobic amino acids in all fish species. It was concluded that hydrophobic amino acids were observed in several peptide sequences with antioxidant properties. Akita et al. (2020) reported studies on the correlation between the content of *Pro*, *Hyp*, and *Ser* and the denaturation temperature of type I collagen with the physiological temperature of marine organisms [141]. The degree of hydroxylation of *Pro* and *Lys* is known to influence the thermal stability of collagen [141]. Chinh et al. (2019) reported amino acid sequences of *Carp* fish scale wastes [142]. From the presented analysis, we can see that the main amino acids in most of the collagens in fish skin are *Gly*, *Pro*, *Ala*, *Glu*, *Hyp*, and *Val*. *Pro* and *Ala* were the most abundant hydrophobic amino acids in all fish species, although there were clear differences. Tryptophan (*Trp*) was not found in all of the species. Table 2 shows the experimental results for the amino acid content of collagen hydrolysates extracted from the skin or swim bladder of the different fish species presented. Regardless of the units of measurement used for reporting these amino acids, *Gly* consistently appears in the highest amounts across all species analyzed. The values are typically expressed in residues per 1000 residues. 

#### 4.2.2. Amino Acids from Crustacean Collagen

*Gly* is found to be the amino acid found in all species studied except *Rhizostoma pulmo*, studied by Cheng et al. (2017), who reported the order *Glu*, *Phe*, and *Leu* [119]. Mequiol et al. (2019) studied *Stomalophus meleagris* and reported the following order: *Gly*, *Glu*, *Pro*, and *Ala* [143]. Aziz et al. (2020) reported values for *Rhopilema hispidum* in the order *Gly*, *Glu*, *Arg*, *Pro*, *Asp*, and *Ala* [144]. Qiu et al. (2020) reported values for amino acids from *Nemopilema nomurai* in the order *Gly*, *Glu*, *Ala*, *Pro*, and *Asp* [145]. Pivnenko et al. (2022) reported amino acids from *Rhopilema asamushi* in the order *Gly*, *Glu*, *Pro*, *Ala*, *Arg*, and *Asp* [146]. James et al. (2023) reported that amino acids were also found in *Rhizostoma pulmo* in the order *Gly*, *Glu*, *Ala*, *Asp*, and *Leu* [147]. Table 3 shows the results of amino acids found in collagen extracts from marine invertebrates: different species of jellyfish, mollusks, and one species of shrimp. Amino acid values are generally reported in mass percent.

Sudirman et al. (2023) studied *Rhopilema esculentum* and reported for amino acids the order *Gly*, *Glu*, *Ala*, and *Pro* [148]. Chiarelli et al. (2023) studied *Stomolophus meleagris* and reported for amino acids the order *Gly*, *Glu*, *Asp*, *Pro*, and *Arg* [149]. Tryptophan (*Trp*) was found to be identified only in *Rhizostoma pulmo* by James et al. (2023) and in *Stomolophus meleagris* by Chiarelli et al. (2023) [147,149]. Hydroxylysine (*Hyl*) was identified only in *Stomalophus meleagris* by Mequiol et al. (2019) and in *Rhopilema asamushi* by Pivnenko et al. (2022) [143,146]. Cysteine (*Cys*) is present in *Nemopilema nomurai*, reported by Qiu et al. (2020); in *Rhizostoma pulmo*, reported by James et al. (2023); and in *Stomolophus meleagris*, reported by Chiarelli et al. (2023) [145,147,149]. It does not show histidine (*His*) in *Rhizostoma pulmo* reported by James et al. (2023) nor *Rhopilema esculentum*, reported by Sudirman et al. (2023) [147,148]. Li N. G. et al. (2018) reported the amino acid content of the mollusk *Corbicula japonicasi*, with values in the order *Glu*, *Asp*, *Leu*, *Lys*, and *Val* [150]. Li X. et al. (2021) reported the amino acid content of the white *shrimp Litopenaeus vannamei* with higher values for *Gly*, *Arg*, *Pro*, and *Ala*. It does not show hydroxylysine (*Hyl*) [151]. The amino acid content of mollusk and shrimp species is much lower than that of jellyfish species. Lima et al. (2019) found that amino acids such as *Asp*, *Gly*, and *Glu* improve wound healing [152]. Hydrophobic amino acids have antioxidant action as they can interact on membrane lipid layers to reach targets and help scavenge radicals [149,152].

## 5. Antioxidant Activity

**Figure 6:**
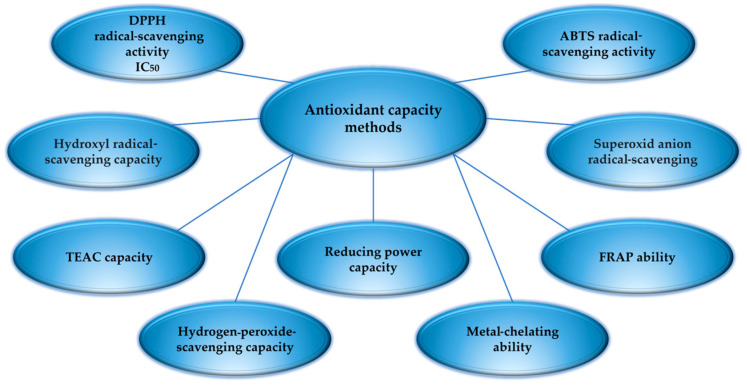
Type of methods used to demonstrate antioxidant activity.

Oxidation is a vital and normal process in vertebrates and humans, whereby free radical species (ROS) are continuously generated in the cellular metabolism. The accumulation of ROS in the body must be kept under control to avoid the diseases they can cause. Oxidative stress is linked to damaging processes such as lipid peroxidation, protein damage, DNA breakdown, or enzyme inactivation. These promote the development of various diseases such as tumor formation or cancer, heart disease, rheumatoid arthritis, or aging. Suo et al. (2022) showed that seventeen ACE inhibitory peptides isolated from the protein hydrolysate of the blue mussel *Mytilus eludis* could be used as natural ingredients in the development of products with antihypertensive functions [153]. Hydrolysates and collagen peptides from fish by-products have demonstrated antioxidant capacity to reduce oxidative processes and can thus be used to produce functional foods. There were researchers like Nikoo et al. (2021) and Nirmal et al. (2022) who reported that certain hydrophobic amino acid sequences provide antioxidant properties as proton or electron donors or as lipid radical scavengers [154,155]. The antioxidant properties of marine collagen peptides and hydrolysates are influenced by several parameters, such as amino acid composition, chain size and length, or residue/chain sequence [150,154]. Chaoting et al. (2020) emphasized the relationship between peptide structure and the antioxidant activity of peptides isolated from proteins [156]. The relationship between structure and the antioxidant activity of peptides derived from marine by-products was presented by Sila et al. (2016) [157]. Other researchers, such as Phadke et al. (2021) and Nirmal et al. (2023), considered that the molecular weight of peptides influences their antioxidant activity [158,159]. The amino acids *Tyr*, *Met*, *Hys*, *Lys*, and *Trp* have strong radical-scavenging activity in oxidative reactions [158]. Nirmal et al. (2023) explained that *Hys* significantly enhances the antioxidant capacity because protonation of the imidazole ring acts as a hydrogen donor [159]. Azizah et al. (2020) showed that another factor influencing the antioxidant activity of peptides besides amino acid composition is the specificity of the protease used in the hydrolytic process [160]. Nirmal et al. (2023) consider the degree of enzymatic hydrolysis important in assessing the antioxidant activity of proteins and peptide derivatives in fish [159]. The types of enzymatic hydrolysis for several types of enzymes described by Teng et al. (2023) are trypsin, papain, pepsin, alcalase, flavourzyme, protamex, and bromlaine. pH values are 2.0–9.0. Temperatures are 37–55 (°C) and the time is 4 h [161]. The antioxidant capacity can be proven by several methods, as shown in Figure 6.

By analyzing and summarizing the data presented in Table 4, we can see that the antioxidant activity of collagen and marine collagen were tested by different methods. The DPPH radical-scavenging activity assay method was used to reveal the antioxidant potential in all of the species exemplified in Table 4 [68,147,160,162,163,164,165,166,167,168,169,170,171,172,173,174,175,176]. The antioxidant activity with the highest percentages obtained by DPPH assay were reported by Zamorano-Apodaca et al. (2020), who extracted peptide fractions from mixed by-product: skins, heads, and skeletons from different fish species (different sharks, mullet, guitarfish, ray, weakfish, snapper, squid, seabass, pompano dolphinfish) [167]. The authors showed that the percentages ranged from 67% to 77% at concentrations of 10 mg/m [167]. Antioxidant activity reported by IC_50_ values that recorded the highest values (IC_50_ = 8.38 mg/mL) was demonstrated by Asaduzzaman et al. (2020), who performed DPPH assays on amino acids extracted from the bone and skin of the mackerel *Scomber japonicas* [68]. For the other species of marine organisms reported in Table 4, the antioxidant potential was also reported by various other specific tests. ABTS scavenging activity is a widely used method for demonstrating the antioxidant activity of extracted collagen peptides [68,160,163,165,168,169,170,171,172,175,176]. The highest values by ABTS assay (83.5% at 2.5 mg/mL) were reported on collagenic peptides extracted from *Cynoscion guatucupa*—stripped weakfish skin—by Lima et al. (2019) [170]. Appreciable values by ABTS assay (81.05% at 500 µg/mL) were also reported by Yang et al. (2020), who analyzed amino acid sequences (*Ala-Thr-Val-Tyr*) with antioxidant potential from the silky shark *Carcharhinus falciformis* [168]. Another method for testing antioxidant potential was hydroxyl radical-scavenging activity [161,164,167,169,170,171,173,176]. By the hydroxyl radical-scavenging method, Zamorano-Apodaca et al. (2020) also reported the highest percentages (from 64% to 85% at concentrations of 10 mg/m) attesting to the antioxidant activity of peptide fractions extracted from mixed by-products: skins, heads, and skeletons from different fish species (different sharks, mullet, guitarfish, ray, weakfish, snapper, squid, seabass, pompano dolphinfish) [167]. The superoxide anion radical-scavenging method was also used to reveal antioxidant activity [161,169,171,172,176]. Using superoxide anion radical-scavenging method on invertebrates, the highest values expressed by IC_50_ (IC_50_ = 1.55 mg/mL) for collagen from whole tissue in the jellyfish *Nemopilema nomurai* were reported by Teng et al. (2023), and in vertebrates the highest values (IC_50_ = 0.91 mg/mL) were reported by Zhang et al. (2019) for the amino acid sequences Pro-Phe-Gly-Pro-Asp from the skin of Japanese Spanish mackerel (*Scomberomorus niphonius*) [161,169]. FRAP ability is a method successfully used in testing the antioxidant potential for collagen compounds in both vertebrates and invertebrates [162,163,165,166,167,175]. The highest values for the antioxidant activity by the FRAP method (1.4% at 2 mg/mL) were reported by Ahmed et al. (2022) for C- and N-terminal amino acid sequences from *Pampus argenteus* skins [162]. Table 4 presents the results of antioxidant activity studies conducted by various researchers on different marine species.

Antioxidant activity can also be assessed by metal-chelating activity [68,165,166]. The highest values in the metal-chelating method were reported by Khesal et al. (2020) for peptide fractions from by-products from *Rutilus frisii kutum* [166]. To attest antioxidant activity, some authors have used four different types of methods; for example, Chotphruethipong et al. (2021) tested the antioxidant activity of hydrolyzed collagen from defatted *Asian sea bass* skin by four methods: DPPH, ABTS, FRAP, and the metal-chelating method [165]. The highest values in the metal-chelating method were reported by Khesal et al. (2020) for peptide fractions from by-products from *Rutilus frisii kutum* [166]. Using the DPPH, ABTS, and hydroxyl and superoxide anion radical-scavenging methods, Zhang et al. (2019) tested the antioxidant potential of amino acid sequences from mackerel (*Scomberomorus niphonius*) skin, and Tao et al. (2018) demonstrated the antioxidant activity of amino acid sequences from *Mustelus griseus cartilage* [169,171]. Also, Yang et al. (2019) reported the antioxidant potential of amino acid sequences from the mollusk *Tergillarca granosa* by four methods: DPPH, ABTS, and the hydroxyl and superoxide anion radical-scavenging methods [176]. Note from Table 4 that antioxidant activity was only reported by DPPH assay for the collagen extracted from the jellyfish *Lobonema smithii* and *Rhopilema hispidum* by Muangrod et al. (2022), and the values for collagen extracted from oral arms are higher than those from whole tissue and respective umbrellas in both jellyfish species [174]. Also, by a single method, ABTS, the antioxidant activity of the peptide fraction < 3 kDa from *Pangasius hypopthalmus* skin was reported by Azizah et al. [160]. Also, Yang et al. (2019) reported the antioxidant potential of amino acid sequences from the mollusk *Tergillarca granosa* by four methods: DPPH, ABTS, and the hydroxyl and superoxide anion-scavenging method [176].

The antioxidant activity is attributed to the amino acid sequences in collagen peptides and varies based on the type of enzymatic hydrolysate used for their separation, as shown in Table 5. Zhao et al. (2018) investigated collagen peptides with antioxidant potential by using pepsin for enzymatic hydrolysis [66]. They isolated collagen from the swim bladders of the Miiuy croaker (*Miichthys miiuy*). Dong et al. (2022) also used pepsin to isolate collagen from the swim bladders of several fish species, including *Miichthys miiuy*, *Labeo rohita*, *Thunnus albacares*, and *Silurus triostegus* [86]. Zhang et al. (2019) identified amino acid sequences from the skin of Lophius litulon, reporting antioxidant activity tested by various specific methods [169].

The antioxidant activity of collagen peptides has also been reported in invertebrates, particularly jellyfish. For instance, James et al. (2023) presented DPPH results for antioxidant activity in *Rhizostoma pulmo* using pepsin hydrolysis [147]. Similarly, De Domenico et al. (2019) reported antioxidant activity in *Rhizostoma pulmo* using TEAC and ABTS assays [177]. The pepsin enzymatic hydrolysis process is the most commonly used. By this process, Chen et al. (2018), who extracted collagen from the scales of *Chanos chanos*, Najafian et al. (2018) from the fish (*Budu*) and Aissaoui et al. (2017) studied the collagen peptides from the small red scorpionfish *Scorpena notate* and used different specific methods to highlight the antioxidant potential of marine collagen [178,179,180]. By enzymatic hydrolysis with collagenases, collagen peptides were extracted from the skin of *Hypophthalmichthys molitrix* by Huang et al. (2023) and from the skin of *Decapterus macarellus* by Herawati et al. (2022) [181,182]. Antioxidant peptides were extracted by enzymatic hydrolysis with collagenases from catfish skin by Ayat et al. (2021) and from lamuru (*Caranx ignobilis*) by Nur et al. (2021), and the antioxidant activity of these peptides was revealed [183,184].

By enzymatic hydrolysis with alkalase, hydrolyzed collagen was extracted from tuna (*Thunnus albacares)* skin by Nurilmala et al. (2020), from *Tilapia* fish bones by Luo et al. (2022), from *Cyprinus carpio* skin by Gonzalez et al. (2022), and from *Theragra chalcogramma* skin by Lee et al. (2022); it showed antioxidant activity tested by specific methods, DPPH, superoxide anion radical scavenging, FRAC ability, TEAC, and ORAC [101,185,186,187]. Table 5 shows the various types of enzymes used in enzymatic hydrolysis and the antioxidant potential of collagen extracts.

Muangrod et al. (2022) and Uptata et al. (2022) studied the antioxidant potential by DPPH, ABTS, and the FRAP ability of peptide fractions extracted from the jellyfish *Lobonema smithii* by enzymatic hydrolysis with alcalase, flavorzyme, and papain hydrolysis [174,175].

Alkalase hydrolysis has been used to obtain collagen extracts by Viji et al. (2019), who demonstrated the antioxidant activity of collagen peptides from the skin and scales of *Cynoglosus arel*, and by Sae-leaw et al. (2018), who extracted such collagen with antioxidant properties from salmon scales, with antioxidant activity tested by DPPH, ABTS, and FRAP ability [188,189].

By enzymatic hydrolysis with papain, collagen was extracted and amino acid fractions with antioxidant properties were studied by Muangrod et al. (2022), who studied the jellyfish *Rhopilema hispidum*; by Jin et al. (2019), who reported data for Sea cucumber *Actinopyga lecanora*; by Islam et al. (2023), who studied amino acid fractions with antioxidant properties from *Sturgeon* fish; and by Chotphruethipong et al. (2021), who extracted collagen hydrolysates from the skin of *Lates calcarifer* [174,190,191,192]. Also, by enzymatic hydrolysis, Iosageanu et al. (2021) extracted collagen peptides from *Hypophthalmichthys molitrix*; Bordbar et al. (2021) extracted collagens from stonefish (*Actinopyga lecanora*); and Qiu et al. (2019) extracted collagen peptides from skipjack tuna (*Katsuwonus pelamis*) scales and conducted studies for the antioxidant potential attested by different methods [193,194,195]. Table 5 presents studies in which the authors present the antioxidant potential of collagen extracts from brown resources attested by different specific methods but emphasize the collagen extraction methods, which, as demonstrated, can influence the extraction yield, the type and purity of extracted components, and the antioxidant properties. We find that most extraction techniques were enzymatic hydrolysis, but also other techniques. Using several types of enzymatic hydrolysis with trypsin, neutrase, protamex, flavorzyme enzymes, trypsin, bromelain, papain, pepsin, and alkalase, different collagen peptides were extracted for which the antioxidant potential was studied. Such were the studies performed by Teng et al. (2023) for collagen from the jellyfish *Nemopilema nomurai*; by Bordbar et al. (2021), Qiu et al. (2019), and Zhang et al. (2022) for gelatin from the skin of skipjack tuna (*Katsuwonus pelamis*); and by Wang et al. (2020) for collagen peptides from the scales of red lip Croaker (*Pseudosciaena polyactis*), who also tested antioxidant activity [161,194,195,196,197]. Other studies for antioxidant activity using enzymatic hydrolysis with multiple enzymes for the extraction of collagen were those reported by Qiu et al. (219) for different collagen peptides extracted from skipjack tuna (*Katsuwonus pelamis*) scales, and by Chel-Guerrero et al. (2020) for peptide fractions extracted from red lionfish (*Pterois volitans* L.), who tested the antioxidant potential using different methods [195,198]. Antioxidant activity was also reported by Jin (2019) for collagen from the sea cucumber *Acaudina molpadioides* and by Zhao et al. (2018) for collagen peptides from the Miichthys miiuy croaker (*Miichthys miiuy*), both studies folding multiple enzymes to obtain collagen compounds [190,199].

Devita et al. (2021) identified amino acids from *Thunnus obessus* skin in different enzymatic hydrolyses (with bromelain, papain, pepsin, and trypsin), and Li et al. (2021) reported mottled duck cartilage collagen in several types of hydrolysis (enzymatic hydrolysis with trypsin, chymotrypsin, and papain) and showed the antioxidant activity of the obtained extracts by DPPH, reducing power, and ABTS [200,201]. Sripokara, P. et al., (2019) using enzymatic hydrolysis with trypsin, reported the antioxidant properties of collagen peptides from starry triggerfish (*Abalistes stellaris*) through several assays: ABTS and DPPH, FRAP ability, and the metal-chelating activity of the hydrolysate sample, which were dose-dependent [202].

Neutrase enzymatic hydrolysis has been used by Bordbar et al. (2021), who extracted collagen from the sea cucumber *Acaudina Molpadioides*; by Qiu et al. (2019), who extracted gelatine and collagen peptides from skipjack tuna (*Katsuwonus pelamis*) scales; and by Zheng et al. (2020), who extracted collagen peptides from the swim bladders of the giant croaker (*Nibea japonica*) and demonstrated their antioxidant activity by different methods, specifically DPPH and ABTS, but also other specific methods [194,195,203]. Using protease enzymatic hydrolysis, Baehaki et al. (2020) extracted collagen peptides from *Channa striata* skin; Kusumaningtyas et al. (2019) extracted collagen hydrolysates from milkfish (*Chanos chanos*) skin; Wu et al. (2018) extracted peptides from collagen hydrolysate obtained from *Salmon* skin; and Vieira et al. (2017) extracted two novel peptides from the head, scales, skin, and blood of sardines (Sardine *(Sardina pilchardus)*, which they tested for their antioxidant activity by specific methods: DPPH, ABTS, and FRAP ability [204,205,206,207].

Using solvents such as diethyl ether extracts to obtain collagens from the skin of the fish *Conger myriaster* and *Anguilla japonica*, Santhanam et al. (2022) were able to isolate collagen peptides with antioxidant activity tested by DPPH assay [208]. Jantaratch et al. (2022) reported amino acid fractions from the skin of *Oreochromis niloticus* in crude enzyme solutions from *Tuna* stomachs, in which they tested antioxidant activity by ABTS and FRAP [209]. Rashid et al. (2023) obtained fish protein hydrolysates from Malaysian fish salami (*Keropok Lekor*) using enzymatic methods by *Lactobacillus casei* fermentation and evaluated their antioxidant and antibacterial activity [210]. Dara et al. (2020) utilized hydrolysis with visceral proteases extracted from the gastrointestinal tracts of fish and demonstrated the antioxidant activity of peptide fractions obtained from *Johnius dusumieris* skin using DPPH, ABTS, and FRAP assays [211].

One other extraction method, hydrolysis of subcritical water for the production of bioactive peptides with antioxidant properties, has been used by Bashir et al. (2020), who identified antioxidant peptides from the mackerel (Scomber Japonicus), and by Ahmed et al. (2018), who identified bioactive peptides from tuna skin collagen [38,212]. Franco et al. (2020) explored the antioxidant properties of collagen with a specific method: DPPH, ABTS, and FRAP assays for collagen extracted from sea bream and sea bass by-products utilized solvents in pulsed electric fields [213]. Yanshole et al. (2019) reported interesting studies on the presence of ovothiol A (OSH) in the lenses of *Sander lucioperca* and *Rutilus rutilus* lacustris fish [214]. Their study shows that high concentrations of OSH levels in fish are seasonally variable [214].

## 6. Antioxidant Applications of Nutraceuticals Based on Collagen, Gelatin, and Collagen Peptides

Nutraceuticals are specialized products consumed with food to provide health benefits beyond basic nutrition. These products come in various forms, like tablets, capsules, powders, and beverages. Functional proteins, a subset of nutraceuticals, are complex mixtures of biologically active proteins that support normal immune function. With a global shift towards healthier lifestyles, there has been a significant investment in nutritional products [215]. According to global reports, the functional protein market is expected to reach USD 7.98 billion by 2026, growing at a CAGR of 6.93% from 2019 [216].

Collagen hydrolysates and peptides derived from marine sources are notable nutraceuticals due to their biological activities.

### 6.1. Anti-Cancer Activity

**Figure 7:**
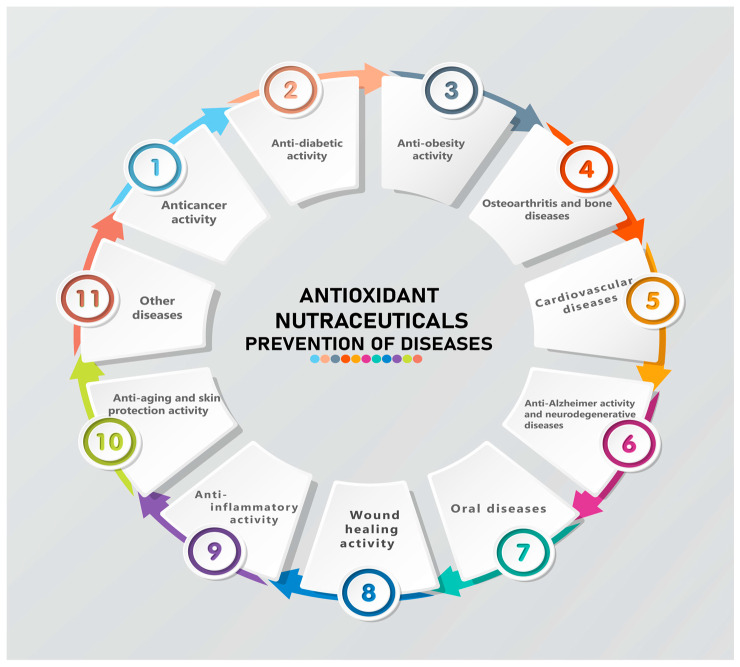
Diseases treated with antioxidant nutraceuticals that have in their compositions peptides and collagen hydrolysates.

Antitumor and antioxidant activity were reported by Mizarpour et al. (2020) on studies done with hydrolysates from *Barred mackerel* skin, which were screened for cytotoxic activity against human MCF-7 cell line cells [217]. Nine fractions obtained by hydrolysis of fish gelatin were tested, of which the F1 fraction was found to have very good antioxidant and anti-carcinogenic activities [217]. Yaghoubzadeh et al. (2019) reported research on hydrolyzed proteins and collagen peptide fractions with molecular masses less than 3 kDa obtained from *Rainbow trout* fish, in which they evidenced antioxidant and anticancer activities in human colorectal carcinoma HCT-16 [218]. Lu et al. (2017) reported the activity of two peptides extracted from cod fish skin that had essential actions in various invasive processes, inhibiting MMP-1, p-ERK, and p-p38 [219]. Ramesh et al. (2021) identified the antitumor cytotoxic activity of *Leji-malides* (A-D), which are unique 24-membered polyene macrolides found in the species *Eudistoma* cf. *rigida* [220]. Meanwhile, Ganesan et al. (2020) and Hu et al. (2012) reported both in vitro and in vivo antitumor activity on HELA and HT-29 cell lines, along with the antioxidant activity of polypeptides with a molecular weight of 20,419 Da extracted from the bivalve mollusca *Archa subcrenata* [215,221]. Their findings showed that the tumor growth inhibition rates of P2 were 26.4%, 41.4%, and 46.4% for hepatoma cells H-22 and 34.0%, 45.8%, and 60.1% for sarcoma cells in S-180 tumor-bearing mice [215,221]. Figure 7 illustrates the common conditions for which nutraceutical antioxidants containing collagen hydrolysates, gelatins, or collagen peptides from marine sources are recommended.

Ganesan et al. (2020) and Beaulieu et al. (2013) reported antitumor activity against various cancer cell lines. They observed mortality rates of 81%, 85%, 89%, and 90% in cell lines BT549 (breast carcinoma), HCT15 (colon carcinoma), A549 (type II lung epithelial), and PC3 (prostate cancer), respectively, at a concentration of 44 mg/mL [215,222]. This activity was attributed to 50 kDa fractions containing 56% of the proteins rich in the amino acids *Thr*, *Pro*, and *Gly*, sourced from the mussel *Mitylus edulis* [215,222]. Additionally, Wali et al. (2019) and Ruiz-Torres et al. (2017) highlighted the specific anticancer properties of coral derivatives. These compounds exhibit anti-inflammatory, anticancer, and antioxidant activities, suggesting potential for cancer treatment [223,224].

### 6.2. Antidiabetic Activity

Xu et al. (2022) reported studies on Gly-Pro-type peptides, containing 4–9 amino acid residues, obtained by enzymatic hydrolysis of tilapia *Oreocchromis niloticus* skin gelatin using seven proteases: papain, bromelain, neutrase, alkalase, protamex, flavorzyme, and trypsin [225]. Some proteases showed differences in peptide release, with the authors concluding that papain released strong dipeptidyl peptidase IV (DPP-IV)-inhibitory peptides to the greatest extent from *Tilapia* fish skin [225]. Wang et al. (2015) reported studies performed on gelatin hydrolysates on different fish from both cold and warm water [226]. They demonstrated that peptide fractions with MW < 1.5 kDa obtained from *Halibut* and *Tilapia* fish presented remarkable DPP-IV inhibitory activity of 38.2% and 51.9%, respectively, at a sample concentration of 1 mg solid/mL and re-performed in vivo antihyperglycemic experiments on streptozotocin-induced diabetic rats, demonstrating improved glucose tolerance. Better results were reported for the amino-acid-rich warm-water fish gelatin from *Tilapia* fish as a more potent antihyperglycemic agent compared to the gelatin hydrolysate from *Halibut*, due to its superior amino acid content [226].

### 6.3. Antiobesity Activity

Wang et al. (2020) reported studies on collagen peptides with molecular weights ranging from 500 to 5000 Da, extracted from an enzymatic hydrolysate of *Walleye Pollock* skin, that had efficient effects against obesity in mice fed a high-fat diet [227]. The results show that collagen peptide extracts from *Walleye pollock* are a potential agent in the development of an adjuvant for the treatment of obesity and associated metabolic diseases [227].

Raksha et al. (2023) reported studies of collagen peptides extracted from the jellyfish *Diplulmaris antarctica* that have action in preventing and treating obesity caused by a high-calorie diet and in curing other pathologies associated with increased oxidative stress [228].

### 6.4. Osteoarthritis and Bone Diseases

Luo et al. (2022) characterized low-molecular-weight collagen peptides, primarily composed of *Gly*, *Ala*, and *Pro*, extracted from *Atlantic salmon* bone. They evaluated these peptides’ effects on chondrocytes induced by interleukin 1β (IL-1β) and assessed their efficacy and safety as anti-osteoarthritis agents through biomarker testing. The goal was to develop a dietary supplement that could delay arthritis development and support anti-inflammatory cartilage regeneration [229].

### 6.5. Cardiovascular Diseases

Hypertension has recently become a major global problem. In recent decades, there has been increasing interest in natural ACE-inhibitory peptides from food. These include by-products from fish skin: collagen, collagen hydrolysates, and collagen peptides, which are an important source of ACE-inhibitory peptides. Cui L. et al. (2023) studied antiplatelet peptides in collagen hydrolysates from silver carp skin that were enriched using macroporous resins. The results showed the yield and antiplatelet activity of the 20% ethanol fraction with an IC_50_ of 2.03 mg/mL, which recommended the use of fish antiplatelet peptides as functional foods [230].

In most cardiovascular diseases, atherosclerosis occurs, which is inflammation of the blood vessels. Liu H. et al. (2022) demonstrated through research on collagen hydrolysates from *Atlantic salmon* fish skin (*Salmo salar*) that they possess potent anti-inflammatory activity, protective activity against endothelial cell injury, antioxidant activity, and anti-platelet aggregation activity in vitro [231]. Also, collagen hydrolysates from *Salmon* fish showed combined effects on the regulation of serum biomarkers of inflammation (IL-6 and TNF-α), on endothelial injury (MCP-1), activating platelets (TXB2 and PF4), and regulating oxidative stress. It can be a dietary supplement for the prevention of atherosclerosis [231]. Abdelhedi et al. (2017) conducted comparative studies on gelatin hydrolysates extracted from black-barred halfbeak (*Hemiramphus far*) hides using different acidic, alkaline, and enzymatic hydrolysis treatments [232]. Their research demonstrated the high antioxidant potential of fish collagen hydrolysates and highlighted the ACE-inhibitory activity of peptides as a promising nutraceutical product for various cardiovascular diseases [232].

Similarly, Aissaoui et al. (2017) studied collagenous hydrolysates from *scorpion* fish (*Scorpaena notata*) red fish heads obtained through enzymatic treatments. The authors showed that these peptides exhibit high inhibitory activity against the angiotensin-I-converting enzyme, with IC_50_ values of 0.98, 1.69, and 1.44 µm. They also concluded that fish by-products can be exploited as nutraceuticals against oxidative stress and hypertension [233]. Thuanthong et al. (2017) and Liu et al. (2019) have highlighted the importance of *Oreochromis niloticus* and *Pinctada fucata martensii* in cardiovascular treatments [234,235]. 

Zhong et al. (2018) optimized the enzymatic hydrolysis process to separate bioactive peptides with ACE-inhibitory activity from sea cucumber (*Stichopus japonicus*) gonads. The peptides were identified showing the highest ACE-inhibitory activity (IC_50_ of 260.22 µM) and cytotoxicity to Caco-2 cells [236]. Zhang et al. (2018) studied peptides extracted from the hydrolysates of jellyfish gonads (*Rhopilema esculentum Kishi*-*nouye*) using neutral proteases. These peptides with the SY amino acid sequence demonstrated both good ACE-inhibitory and antioxidant activities [237]. This purified dipeptide is recommended as a functional food material for its antioxidant properties and ACE-inhibitory activity [237].

### 6.6. Anti-Alzheimer’s Activity and Neurodegenerative Diseases

Alzheimer’s disease is a neurodegenerative disease that occurs due to the progressive loss of neurons. Abuine et al. (2019) and Choi et al. (2015) showed that the prevalence of neurodegenerative diseases increased with increasing life expectancy [238,239]. Abuine et al. (2019) and Lee et al. (2015) reported as mechanisms of action the inhibitory effect of β-secretase attributed to the peptide sequence QGYRPLRGPEFL [238,240].

The neuroprotective effect and antioxidant activity of protein extracts from the skin of grass carp (*Ctenopharyngodon idella*) has also been shown by Abuine et al. (2019) and Cai et al. (2015), who showed that PYSFK-, GFGPZL-, and VGGRPP-type peptides showed important neuroprotective activity [238,241]. Abuine et al. (2019) and Xu et al. (2015) showed neuroprotective effects in Alzheimer’s disease with the presentation of collagen peptides from catfish (*Oncorhynchus keta*) [238,242]. Ganesan et al. (2020) and Pangestuti et al. (2013) reported neuroprotective effects against Ab42-induced neuronal death in PC12 cells by collagen peptides extracted from the sea horse (*Hippocampus trimaculatus*): GTZDZLDK [215,243].

### 6.7. Oral Diseases

One of the most prevalent diseases of the oral cavity is oral mucosal ulcers, which manifest as severe burning pain and difficulty chewing, drinking, and even speaking. Gao et al. (2022) evidenced the role of collagens from marine resources in the healing of oral cavity wounds [244]. They demonstrated that low-molecular-weight collagen peptides from *Tilapia* fish skin play a role in the healing of traumatic oral ulcers in rats [244]. Xu et al. (2021) proved through research on periodontal membrane cell culture experiments of hydrolyzed *Tilapia* fish collagen that it has the function of regenerating periodontal tissue in vitro [245]. *Tilapia* fish collagen has been used in the production of composite membranes as nanofibers, together with bioactive glass and chitosan. Zhou, T et al. (2017) made a biomimetic fish collagen/bioactive glass/chitosan (Col/BG/CS) nanofiber composite membrane to study the biological effects on human periodontal ligament cells (HPDLCs) [246]. The results of Tang et al. (2015) suggested that tilapia scale collagen might be a potential alternative to type I collagen for use in oral diseases [247]. Liu C et al. (2015) suggested for the first time that hydrolyzed tilapia fish collagen (HFC) can be used for periodontal tissue regeneration and is a promising bioactive ingredient for biomaterials used in alveolar bone regeneration [248].

### 6.8. Wound Healing Activity

Chotphruethipong et al. (2021) proved antioxidant and anti-inflammatory activities in wound healing of sea bass (*Lates calcarifer*) collagen hydrolysates conjugated with epigallocatechin gallate through the inhibition of nitric oxide production and tumor necrosis factor-α in RAW264.7 cells [192]. Also, Chotphruethipong et al. in 2021 reported studies on two collagenous peptides (PO and POG) isolated from the skin of *Asian sea* bass (*Lates calcarifer*), which showed antioxidant effects [249]. Sivarman et al. (2021) showed that the collagen peptide induces cell growth and migration of fibroblast cells and facilitates the wound healing process. They recommended the use of these peptides as a functional ingredient for nutraceuticals used in wound healing [250]. Chen et al. (2019) demonstrated the existence of collagen peptides from a collagen sponge extracted from the bladder of *Nibea japonica* with the GAPO sequence, which produced accelerated wound healing [92]. Mice treated with sponge collagen had significantly reduced interleukins. These have potential applications for wound healing.

### 6.9. Anti-Inflammatory Activity

Sivaraman et al. (2021) reported obvious anti-inflammatory effects generated by peptide fractions with molecular weights of 1–3 kDa extracted from the skin of the fish *Clarias batrachus* and *Pangasius pangasius*. Peptide fractions from these two fish species showed a suppression of inflammatory proteins (TNF-α, IL-6, NF-κB, and p-IκB). Due to these properties, the collagen hydrolysates of these fish species can be functional foods, and purified fractions can be used as nutraceuticals with anti-inflammatory properties [250].

### 6.10. Anti-Aging and Skin Protection Activity

Skin aging occurs under the action of intrinsic (e.g., aging) and extrinsic (e.g., smoking and UV) factors. UV irradiation consists of UV-A, UV-B, and UV-C. Fu et al. (2022) showed that UV-B radiation is responsible for the largest proportion of photoaging, mainly by inducing epidermal and superficial dermal damage [251]. They demonstrated that UV-B irradiation can cause excessive production of reactive oxygen species (ROS) and a range of skin damage through several signaling pathways, such as the stimulation of mitogen-activated protein kinase (MAPK) activity [251]. Xia et al. (2021) reported on natural bioactive peptides with anti-aging effects. They detailed the molecular mechanisms involved [252]. Maia Campos et al. (2021) evaluated the clinical efficacy of low-dose oral supplements of fish cartilage hydrolysate [253]. After a 90-day treatment period, there was a significant reduction in wrinkles and an increase in dermis echogenicity compared to the placebo and baseline values [253].

Table 6 systematizes the biological activities of collagen hydrolysates, collagen peptides, and amino acid sequences from various marine organisms with results in the treatment of various diseases.

### 6.11. Other Diseases

#### 6.11.1. Anti-Allergic Activity

Wang et al. (2020) studied the by-products of Atlantic salmon (*Salmo salar*) and extracted enzymatic hydrolysate collagenic peptides from them [254]. Their research identified six fractions, with fraction C6 demonstrating the strongest antiallergic activity [254]. Additionally, they isolated a novel eleven-amino-acid peptide, TPEVHIAVDKF, which showed antiallergic properties. This study suggests that *Atlantic salmon* by-products could be a valuable source of new ingredients for food and pharmaceutical products aimed at managing food allergies [254].

#### 6.11.2. Treating Malnutrition

Salindeho et al. (2022) reported studies on fish scale peptides mixed with hydroxyapatite and chitin and showed that each component has multiple beneficial properties for the human body as an antioxidant, in the treatment of malnutrition, as a hypocholesterol-lowering agent, and in bone metabolism [255].

#### 6.11.3. Iron Deficiency Treatment

Wu et al. (2015) reported studies on Pacific cod gelatin and showed that several amino acids can be bound by iron ions. This study suggests a potential application of gelatin-derived peptides as novel carriers to combat iron deficiency [256].

## 7. Conclusions

The present study highlights the significance of marine-derived collagen compounds and marine resources for obtaining collagen and collagen peptides from both invertebrates and vertebrates. Based on the literature, enzymatic hydrolysis of collagen, which releases peptides and peptide moieties, is an efficient method for obtaining natural antioxidant compounds from marine sources. Various tests, such as DPPH and ABTS scavenging activity, hydroxyl and superoxide anion radical-scavenging activity, FRAP capacity, and metal-chelating activity, have demonstrated antioxidant activity. However, there are limited data on the beneficial effects of these isolated collagen peptide fractions on human health in in vivo studies for alternative treatments. This review has shown that marine collagen antioxidants from different vertebrate and invertebrate species can be involved in treatments for cancer, diabetes, obesity, osteoarthritis, cardiological conditions, and Alzheimer’s disease. Additionally, collagen antioxidants are used in bone tissue regeneration and osteoarthritis, antihypertensive and neurodegenerative diseases, oral and dental diseases, cell regeneration against oxidative stress, skin lesion healing and protection, anti-inflammatory and anti-allergic responses, and iron deficiency treatments.

The global consumption of marine products has increased due to the use of marine by-products, which are rich in bioactive components that enhance human health by creating novel nutraceutical compounds with antioxidant properties. The objective of fully exploiting marine resources can also be achieved through the efficient and effective use of fish by-products (skin, bones, scales, fins, and fish heads) which contain significant amounts of collagen and collagen peptides. This paper supports the growing utilization of marine antioxidant biocompounds. However, it is often unclear what kind of water or cultural environment certain by-products originate from, raising concerns about the efficacy and safety of these nutraceuticals for human health. Therefore, further research is needed to identify barriers and ensure successful production of antioxidant nutraceuticals from marine resources in the food, pharmaceutical, and biomedical industries.

## Figures and Tables

**Table 1:** Marine collagen isolated from marine vertebrates and invertebrates. Marine species, tissue from marine organism, extraction method, extraction time and yield, physicochemical methods of characterization, and type of isolated collagen.

Marine Sources Species	Tissue	Type of Extraction Method/Time	Collagen Content %	Methods for Characterization	Type of Collagen	References
Vertebrates						
Alu alu (*Sphyraena* sp.)	Skin	ASC 72 h; (4 °C);	6.77	SDS-PAGE, SEM, DSC, XRD, ATR-FTIR,	I	[75]
Asian sea bass (*Lates calcarifer*)	Skin	ASC 24 h; (4 °C);	59.31	UV spectra, SDS-PAGE, SEM, FTIR, XRD,	I	[47]
Seer fish (*Scomberomorus commerson*)	Skin	ASC 24 h; (4 °C);	58.21	FTIR, XRD, SDS-PAGE, UV spectra, SEM	I	[47]
Yellowfin tuna (*Thunnus albacares*)	Skin	ASC 24 h; (4 °C);	61.26	UV spectra, FTIR, XRD, SEM	I	[47]
Round goby (*Neogobius melanostomus*)	Skin	ASC 48 h; (4 °C);	10	SDS-PAGE, FTIR	I	[76]
Silver catfish (*Pangasius* sp.)	Skin	PSC 48 h; (4 °C);	26.4	Solubility, FTIR, SEM, rheology	I	[77]
Unicornfish (*Naso reticulatus*)	Bone	ASC 72 h; (4 °C);	0.4	UV spectra, SEM, SDS-PAGE, FTIR, XRD	I	[78]
Parrotfish (*Scarus sordidus*)	Scale	ASC 48 h; (4 °C); PSC 48 h; (4 °C);	1.17 1	ATR-FTIR, SDS-PAGE, UV-Spectra, XRD, DSC, Solubility	I	[79]
Megalonibea (*Megalonibea fusca)*	Swim bladder	ASC 48 h; (4 °C); PSC 48 h; (4 °C);	33.4 84.8	Amino acid analysis, SDS-PAGE, XRD, UV spectra, FTIR, SEM, zeta potential,	I	[80]
Totoaba (*Totoaba macdonaldi*)	Swim bladder	PSC 24 h; (4 °C);	65	SDS-PAGE, UV spectra, amino acid analysis, FTIR, XRD, zeta potential	I	[81]
Sea eel (*Muraenesox cinereus*)	Swim bladder	PSC 48 h; (24 °C);	93.7	SDS-PAGE, FTIR, SEM, UV spectra	I	[82]
Blue shark (*Prionace glauca*)	Cartilage	PSC 24 h; (4 °C);	7.69	SDS-PAGE, SEM, UV spectra, amino acid analysis	II	[83]
Lizardfish (*Saurida tumbil*)	Bone	ASC 72 h; (4 °C);	1.73	UV spectra, ATR-FTIR, SDS-PAGE, XRD, DSC,	I	[84]
Skipjack tuna (*Katsuwonus pelamis*)	Tail tendon	ASC 72 h; (4 °C); PSC 27 h; (4 °C);	8.67 12.04	SDS-PAGE, viscosity, SEM, FTIR, DSC	I	[85]
Grass carp (*Ctenopharyngodon idella*)	Swim bladder	PSC 48 h; (4 °C);	38.9	FTIR, UV spectra, DSC, SDS-PAGE,	I	[86]
Greenland halibut (*Reinhardtius hippoglossoides*)	Skin	ASC 96 h; (4 °C);	3.8	SDS-PAGE, FTIR, amino acid analysis, SEM-EDX, DSC	I	[87]
Catfish (*Silurus triostegus*)	Skin	ASC 72 h; (4 °C); PSC 72 h; (4 °C);	2.6; 8.24	SDS-PAGE, FTIR, HPLC, SEM, solubility, viscosity	I	[88]
Dusky grouper (*Epinephelus marginatus*)	Scale	ASC 72 h; (8 °C); PSC 24 h; (8 °C);	0.39 1.5	SDS-PAGE, SEM, FTIR, XRD, TGA	I	[89]
Shark (*Prionace glauca*)	Cartilage	ASC 48 h; (4 °C); PSC 48 h; (4 °C);	0.15 3.5	SDS-PAGE, UV spectra, DSC, FTIR, amino acid composition, rheology	I	[62]
Sturgeon fish (*Huso huso*)	Skin	ASC 48 h; (4 °C); PSC 48 h; (4 °C);	9.98 9.08	SDS-PAGE, amino acid analysis, FTIR, SEM, DSC	I	[90]
Sturgeon (*Acipenser baerii*)	Gelatin from cartilage	ASC 7 h; pH 9 (45 C);	28.8	SDS-PAGE, UV and FTIR, amino acid composition, zeta potential	I	[91]
Giant croaker (*Nibea japonica*)	Swim bladder	ASC 24 h; (4 °C); PSC 8 h; (4 °C);	11.3 15.35	SDS-PAGE, FTIR, SEM, amino acid analysis	I	[92]
Bigeye tuna (*Thunnus obesus*)	Skin	ASC 72 h; (4 °C); PSC 72 h; (4 °C);	13.5 16.7	SDS-PAGE, FTIR, amino acid composition, zeta potential	I	[93]
Nile tilapia (*Oreochromis niloticus*)	Scale	ASC 72 h; (10 °C); PSC 72 h; (10 °C);	0.77 0.71	SDS-PAGE, amino acid composition, FTIR spectra, thermal denaturation temperature, zeta potential		[94]
Silver catfish (*Pangasius* sp.)	Skin	ASC 24 h; PSC 24 h;	4.27 2.27	FTIR, SEM, solubility	I	[95]
Bonylip barb fish (*Osteochilus vittatus*)	Skin	PSC 8 h; (4 °C);	6.18	FTIR, HPLC, viscosity, denaturation values	I	[96]
Hammerhead shark (*Sphyrna lewini*)	Cartilages	ASC 72 h; (4 °C);	5.64	SDS-PAGE and peptide mapping, amino acid composition, FTIR, viscosity of collagen, solubility	I	[97]
Mackerel (*Scomberomorous niphonius*)	Skin, bone	ASC; PSC ASC; PSC	58.62; 14.43 13.68; 3.48	SDS-PAGE, amino acid composition, FTIR, solubility	I	[98]
Monkfish (*Lophius litulon*)	Swim Bladders	ASC 48 h; (4 °C); PSC 48 h; (4 °C);	4.27 9.54	SDS-PAGE. amino acid analysis, mass spectrum UV absorption, solubility	I	[99]
Miiuy croaker (*Miichthys miiuy*)	Scales	ASC 48 h; (4 °C); PSC 48 h; (4 °C);	0.64 3.87	SDS-PAGE, amino acid composition, FTIR, UV, viscosity, solubility, zeta potential, SEM	I	[100]
Invertebrate						
Sponge (*Chondrosia reniformis*) (Poriferans)	Sponge tissue	Enzymatic digestion 72 h; (27 °C);	3.4	Amino acid analysis, glycosaminoglycan quantification, viscosity, thermal stability	IV	[105]
Marine sponges (*Chondrilla caribensis*)	Sponge tissue	Four protocols ASC, WSC, PSC Lyophilized marine sponge 24 h	P1 (39.2%); P2 (47.2%), P3 (48.2%); P4 (48.3%)	SEM micrographs; FTIR spectroscopy; circular dichroism	I	[106]
Sponge (*Chondrosia reniformis*) (Poriferans)	Sponge tissue	Enzymatic digestion 24 h (37 °C)	19	Glycosaminoglycan quantification, TEM	IV	[107]
Common starfish (*Asterias rubens*) (Echinoderms)	Body wall	ASC + PSC 48 h; (4 °C);	1.44	UV spectra, FTIR, SDS-PAGE, amino acid analysis, SEM, solubility	I	[111]
Starfish (*Asterias pectinifera*) (Echinoderms)	Body wall	UAC 1 h;	3.8	Amino acid analysis, zeta potential, TEM	I	[112]
Sea cucumber (*Holothuria cinerascens*) (Echinoderms)	Body wall	ASC + PSC 72 h; (4 °C);	72.2	SDS-PAGE, FTIR, UV spectra, amino acid composition	I	[113]
Sea cucumber (*Apostichopus japonicus*)	Body wall	PSC 12 h; (4 °C);	72 h	SDS-PAGE sea cucumber collagen fibrils are heterotypic. Included two clade A fibrillar collagens, one clade B fibrillar collagen, and two FACIT collagens.	Heterotypic	[114]
*Stomolophus meleagris*	Body wall	NaOH for extraction for crude gelatin 24 h and 4 °C	10.49	SDS-PAGE, FT-IR and ^1^H-NMR spectra, amino acid composition		[115]
Jellyfish (*Rhopilema esculentum*) (Coelenterate)	Umbrella	PSC 72 h; (4 °C);	4.31	% I SDS-PAGE, FTIR	I	[116]
Jellyfish (*Catostylus mosaicus*) (Coelenterate)	Umbrella, oral arm	ASC 72 h; (4 °C); ASC 72 h; (4 °C);	1.46 2.24	SDS-PAGE, ATR-FTIR, amino acid analysis, raman spectra	I	[117]
Jellyfish (*Acromitus hardenbergi*)	Bell and oral arms	ASC PSC	0.09–0.29 0.29–0.39 of lyophilized collagen	Physicochemical analysis, amino acid composition	I	[118]
Jellyfish (*Rhopilema esculentum*) (Coelenterate)	Tissue	ASC 72 h; (4 °C); PSC 24 h; (4 °C);	0.12 0.28	SDS-PAGE, FTIR, SEM, amino acid analysis	I	[119]
Blue mussel (*Mytilus edulis*) byssus	Body wall	PSC 4 h; (50 °C);	1.38	SDS-PAGE, protein determination, RP-HPLC, GP-HPLC	IV	[120]
Byssus of Chilean mussels (*Mytilus Chilensis*) (mollusk)	Mussels	ASC 24 h; (80 °C); PSC 24 h; (80 °C);	1.8 7.6	SDS-PAGE, amino acid analysis	I	[121]
Mantis shrimp (*Miyakella nepa*) (Crustacean)	Muscles	PSC 72 h; (4 °C);	0.478	SDS-PAGE, FTIR, solubility	I	[122]
Surf clam shell (*Coelomactra antiquata*)		GSC PSC	0.59 3.7	SDS-PAGE, FT-IR spectra, scanning electron microscopy (SEM), amino acid analysis	I	[123]
Jumbo squid (*Dosidicus gigas*)	Fins Mantle Arms	PSC	17.85 17.65 361.68	SDS-PAGE, FT-IR spectra, amino acid composition, OFF GEL electrophoresis	I	[124]

**Table 2:** Amino acids from fish collagen from skin and other subproducts.

Amino Acids	*Thunnus albacares*	Tilapia Collagen	*Rutilus frisii kutum*	*Pangasianodon hypophthalmus*	*Oreochromis niloticus*	*Channa* *striata*	*Pagrus* *major*	*Paralichthys olivaceus*	*Cyprinus* *carpio*	*Totoaba macdonaldi*	*Totoaba macdonaldi*
Tissue	Skin *	Skin **	Skin *	Skin *	Skin *	Skin *	Skin **	Skin **	Skin **	TSBC **	Swim bladder ***
Essential amino acids (EAAs)											
*Arginine (Arg)*	92.16 ± 2.97	7.91	70.8	53	52	56 ± 3	65.1 ± 1.0	60.6 ± 1.9	51.7 ± 0.9	59 ± 4.47	11.58 ± 0.37
*Cysteine (Cys)*	0.07 ± 0.00	-	-	2	2	2 ± 1	0.9 ± 0.5	0.7 ± 0.3	1.5 ± 0.1	-	0.03 ± 0.01
*Glutamic acid (Glu)*	97.89 ± 0.43	10.16	81.1	75	71	74 ± 4	57.9 ± 1.9	57.0 ± 0.4	74.0 ± 2.1	101 ± 1.95	9.61 ± 0.14
*Glycine (Gly)*	217.22 ± 1.32	23.60	182.5	334	332	307 ± 7	370.7 ± 2.9	395.6 ± 1.3	318.2 ± 3.6	309 ± 3.15	29.19 ± 0.31
*Histidine (His)*	12.70 ± 0.05	1.10	8.8	6	7	6 ± 1	5.2 ± 0.1	6.0 ± 1.0	10.7 ± 3.2	5 ± 0.34	0.47 ± 0.02
*Isoleucine (Iso)*	14.26 ± 0.15	1.40	10.7	6	7	9 ± 2	9.3 ± 0.8	0.9 ± 0.8	13.3 ± 0.8	5 ± 0.22	0.63 ± 0.01
*Leucine (Leu)*	28.28 ± 0.21	2.85	21.1	24	22	28 ± 3	25.1 ± 0.9	22.2 ± 0.4	26.8 ± 1.1	20 ± 0.08	1.94 ± 0.05
*Lysine (Lys)*	35.37 ± 0.23	3.19	31.1	26	28	31 ± 2	24.3 ± 1.0	24.1 ± 0.8	29.4 ± 1	31 ± 1.63	2.52 ± 0.04
*Hydroxylysine (Hyl)*	-	-	-	7	6	6 ± 1	-	-	-	5 ± 0.22	0.28 ± 0.02
*Methionine (Met)*	6.29 ± 0.13	-	14.8	33	34	12 ± 1	10.7 ± 1.5	10.8 ± 0.2	12.8 ± 1.1	7 ± 0.47	1.36 ± 0.03
*Phenylalanine (Phe)*	20.75 ± 0.15	1.73	20.6	14	16	18 ± 2	14.4 ± 1.8	13.4 ± 1.1	14.4 ± 0.5	19 ± 0.54	1.61 ± 0.03
*Proline (Pro)*	114.86 ± 0.45	11.01	89.6	111	112	126 ± 5	89.5 ± 0.7	77.85 ± 0.0	109.9 ± 2.1	122 ± 1.33	12.10 ± 0.13
*Hydroxiproline (Hyp)*	87.38 ± 0.60	8.92	-	81	83	94 ± 5	43.3 ± 0.5	46.5 ± 0.7	70.2 ± 2.1	83 ± 1.43	5.43 ± 0.05
*Threonine (Thr)*	40.00 ± 1.81	3.28	20.6	26	24	22 ± 2	28.0 ± 0.5	26.8 ± 0.8	26.6 ± 0.7	13 ± 2.62	1.76 ± 0.06
*Tryptophan (Trp)*	-	-	-	-	-	-	-	-	-	-	-
*Tyrosine (Tyr)*	4.42 ± 0.07	-	4.4	2	1	5 ± 1	5.1 ± 0.6	4.0 ± 0.4	5.6 ± 2.2	2 ± 0.19	0.47 ± 0.03
*Valine (Val)*	25.64 ± 0.15	2.36	-	25	26	26 ± 1	17.1 ± 3.4	18.0 ± 0.9	24.1 ± 0.8	16 ± 0.3	1.58 ± 0.04
Non-essential amino acids (NEAAs)											
*Alanine (Ala)*	111.78 ± 2.58	11.78	73.1	96	98	89 ± 5	164.5 ± 1.3	157.1 ± 1.4	119.0 ± 1.2	132 ± 1.27	12.26 ± 0.13
*Aspartic acid (Asp)*	55.40 ± 0.54	5.59	42.1	46	47	54 ± 3	34.0 ± 0.6	33.5 ± 0.5	53.1 ± 1.1	52 ± 1.48	5.13 ± 0.05
*Serine (Ser)*	35.53 ± 0.25	3.54	34.8	33	32	35 ± 2	34.9 ± 0.7	37.2 ± 0.3	38.5 ± 0.7	23 ± 0.69	2.02 ± 0.08
Reference	[134]	[135]	[136]	[137]	[137]	[138]	[139]	[139]	[140]	[81]	[81]

TSBC (totoaba swim bladder collagen); (Results are expressed in * %; ** Residues/1000 residues, *** g/100 g amino acid).

**Table 3:** Amino acids from marine crustacean collagen from complete organisms.

Amino Acids	*Rhizostoma pulmo*	*Stomalophus meleagris*	*Rhopilema* *hispidum*	*Nemopilema nomurai*	*Rhopilema asamushi*	*Rhizostoma pulmo*	*Rhopilema* *esculentum*	*Stomolophus* *meleagris*	*Corbicula japonica* (Mollusk)	*Litopenaeus vannamei* (Shrimp)
Tissue	Whole body *	Whole body *	Whole body *	Whole body *	Whole body *	Whole body *	Whole body **	Whole body *	Whole body *	Whole body ***
Essential amino acids (EAAs)										
*Arginine (Arg)*	20	52	8.84	3.87	7.2 ± 0.5	5.63	55.87	8.3 ± 0.1	4.25	11 ± 0.48
*Cystine (Cys)*	13	-	4.87	-	0.6 ± 0.1	-	2.4	1.1 ± 0.0	0.36	1.10 ± 0.05
*Glutamic acid (Glu)*	152	98	10.42	9.98	10.8 ± 1.2	13.46	103.21	13.1 ± 0.1	14.48	2.78 ± 0.13
*Glycine (Gly)*	53	309	19.21	34.82	28.8 ± 1.6	29.34	324.84	19.7 ± 0.3	6.94	15.3 ± 0.66
*Histidine (His)*	56	2	3.28	0.29	3.0 ± 0.2	-	-	1.7 ± 0.1	3.66	1.14 ± 0.05
*Isoleucine (Iso)*	55	22	2.98	1.88	2.1 ± 0.4	-	11.78	2.6 ± 0.0	3.95	1.55 ± 0.06
*Leucine (Leu)*	91	34	3.79	3.09	3.2 ± 0.4	6.35	30.68	3.7 ± 0.0	8.97	2.7 ± 0.11
*Lysine (Lys)*	69	38	3.22	2.96	4.2 ± 0.6	4.62	30.22	4.3 ± 0.2	7.73	3.92 ± 0.12
*Hydroxylysine (Hyl)*	-	27	-	-	2.2 ± 0.5	-	-	-	-	-
*Methionine (Met)*	46	4	2.80	0.21	0.7 ± 0.1	-	8.57	1.3 ± 0.0	0.16	0.9 ± 0.04
*Phenylalanine (Phe)*	93	10	2.16	0.9	2.2 ± 0.4	-	14.86	1.5 ± 0.0	3.74	1.53 ± 0.05
*Proline (Pro)*	39	82	6.93	8.16	8.4 ± 1.0	2.97	95.63	8.7 ± 0.2	6.43	6.6 ± 0.31
*Hydroxiproline (Hyp)*	-	40	5.84	6.33	3.9 ± 0.7	4.82	46.86	6.9 ± 0.1	2	0.031 ± 0.001
*Threonine (Thr)*	50	35	4.30	3.55	3.1 ± 0.3	3.18	27.33	3.9 ± 0.0	3.26	1.89 ± 0.07
*Triptophan (Trp)*	-	-	-	-	-	4.72	-	0.3 ± 0.0	-	1.12 ± 0.04
*Tyrosine (Tyr)*	76	6	1.71	0.11	3.9 ± 0.3	1.77	7.33	1.1 ± 0.0	3.90	2.43 ± 0.08
*Valine (Val)*	49	35	3.23	2.74	3.1 ± 0.5	2.8	22.21	2.6 ± 0.0	7.68	2.17 ± 0.09
Non-essential amino acids (NEAAs)										
*Alanine (Ala)*	39	82	6.11	8.36	7.8 ± 0.3	10.38	100.74	6.7 ± 0.0	5.22	5.98 ± 0.26
*Aspartic acid (Asp)*	32	79	6.78	7.38	7.2 ± 0.8	10.91	76.86	9.0 ± 0.1	10.51	1.62 ± 0.06
*Serine (Ser)*	67	45	3.43	4.89	4.7 ± 0.5	-	29.85	3.3 ± 0.0	5.99	1.64 ± 0.06
Reference	[119]	[143]	[144]	[145]	[146]	[147]	[148]	[149]	[150]	[151]

Results are expressed in; * %; ** Residues/1000 residues; *** mg/g dry weight.

**Table 4:** Antioxidant activity of various marine species due to the composition of different amino acid sequences tested by different physicochemical methods.

Fish Species	Source	Amino Acid Sequence/Amino Acid Fraction	Assay Method and Scavenging Rates Results/IC_50_ Values						References
			DPPH Radical-Scavenging Activity	ABTS-Scavenging Activity	(OH−) Hydroxyl Radical-Scavenging Activity	(O_2_^−^) Superoxide Anion Radical-Scavenging Activity	FRAP	Metal-Chelating Activity	
*Pangasius hypopthalmus*	skin	Peptide fraction < 3 kDa	-	45.98% at 50 µmol/g	-	-	-	-	[160]
*Lutjanus erythropterus*	skin	Amino acid sequences with C- and N-terminals	39.57% at 2 mg/mL	-	-	-	0.8% at 2 mg/mL	-	[162]
*Pampus argenteus*	skin	Amino acid sequences with C- and N-terminal	40.89% at 2 mg/mL	-	-	-	1.4% at 2 mg/mL	-	[162]
*Thunnus albacares*	skin	Peptides (45–245 kDa)	47.1% at 10 mg/mL	43.7% at 10 mg/mL	-	-	0.115% at 10 mg/mL	-	[163]
*Acipenser baerii*	cartilages	*Gly-Glu-Tyr-Gly-Phe-Glu*	IC_50_ = 1.27 mg/mL	-	IC_50_ = 1.16 mg/mL	-	-	-	[164]
		*Pro-Ser-Val-Ser-Leu-Thr*	IC_50_ = 1.05 mg/mL	-	IC_5_ = 0.97 mg/mL	-	-	-	
		*Gly-Ile-Glu-Leu-Phe-Pro-*	IC_50_ = 1.38 mg/mL	-	IC_50_ = 1.63 mg/mL	-	-	-	
Asian sea bass	skin	Amino acid (74 Da–10.175 Da)	8.97 mmol TE/g	650.20 mmol TE/g	-	-	0.36 mmol TE/g	20.94 mmolTE/g	[165]
*Rutilus frisii kutum*	by-products	Peptide fraction	67% at 600 g/L	-	-	-	0.78% at 600 g/L	61.33% at 600 g/L	[166]
Various fish species (different sharks, mullet, guitarfish, ray, weakfish, snapper, squid, seabass, and pompano dolphinfish)	mixed by-products: skins, heads, skeletons	F1 (≥30 kDa)	75% at 10 mg/mL	-	64% at 10 mg/mL	-	0.127% at 10 mg/mL	-	[167]
		F2 (10–30 kDa)	75% at 10 mg/mL;	-	78% at 10 mg/mL	-	0.226% at 10 mg/mL	-	
		F3 (5–10 kDa)	68% at 10 mg/mL	-	84% at 10 mg/mL	-	0.247% at 10 mg/mL	-	
		F4 (1–5 kDa)	67% at 10 mg/mL	-	85% at 10 mg/mL	-	0.309% at 10 mg/mL	-	
		F5 (≤1 kDa)	77% at 10 mg/mL	-	85% at 10 mg/mL	-	0.345% at 10 mg/mL	-	
*Scomber japonicus*	bone	Amino acid (<1650 Da)	IC_50_ = 8.38 mg/mL	IC_50_ = 2.61 mg/mL	-	-	-	IC_50_ = 7.27 mg/mL	[68]
	skin	Amino acid (<1650 Da)	IC_50_ = 7.58 mg/mL	IC_50_ = 2.50 ± 0.05 mg/mL	-	-	-	IC_50_ = 7.01 mg/mL	
*Carcharhinus falciformis*	shark skin	Peptide fraction F19	45.63% at 1 mg/mL	-	-	-	-	-	[168]
	shark skin	*Ala-Thr-Val-Tyr*	-	81.05% at 500 µg/mL	-	-	-	-	
*Scomberomorous niphonius*	skin	*Pro-Phe-Gly-Pro-Asp*	IC_50_ = 0.80 mg/mL	IC_50_ = 0.86 mg/mL	IC_50_ = 0.81 mg/mL	IC_50_ = 0.91 mg/mL	-	-	[169]
		*Tyr-Gly-Pro-Met*	IC_50_ = 0.72 mg/mL	IC_50_ = 0.82 mg/mL	IC_50_ = 0.88 mg/mL	IC_50_ = 0.73 mg/mL	-	-	
*Cynoscion guatucupa*—stripped weakfish skin	skin	Peptide sequence with 1263.58 Da	65.2% at 2.5 mg/mL	83.5% at 2.5 mg/mL	69.7% at 2.5 mg/mL	-	-	-	[170]
*Mustelus griseus*	cartilages	*Gly-Ala-Glu-Arg-Pro*	IC_50_ = 3.73 mg/mL	IC_50_ = 0.10 mg/mL	IC_50_ = 0.25 mg/mL	IC_50_ = 0.09 mg/mL	-	-	[171]
		*Gly-Glu-Arg-Glu-Ala-Asp*	IC_50_ = 1.87 mg/mL	IC_50_ = 0.05 mg/mL	IC_50_ = 0.34 mg/mL	IC_50_ = 0.33 mg/mL	-	-	
		*Ala-Glu-Val-Gly*	IC_50_ = 2.30 mg/mL	IC_50_ = 0.07 mg/mL	IC_50_ = 0.06 mg/mL	IC_50_ = 0.18 mg/mL	-	-	
*Katsuwonus pelamis*	bone	*Ser–Ser–Gly–Pro–Pro–Val-Pro–Gly–Pro–Met–*	IC_50_ = 3.149 mM	IC_50_ = 9.489 mM	-	IC_50_ = 3.803 mM	-	-	[172]
*Oreochromis niloticus*	scales	S1 (66,430 Da) Peptides	29.58% at 1 mg/mL;	-	52.26% at 1 mg/mL	-	-	-	[173]
		S2 (1335 Da) Peptides	24.30 %at 1 mg/mL	-	43.54% at 1 mg/mL	-	-	-	
*Rhizostoma pulmo*	whole tissue	-	38.05% at 5 mg/mL	-	-	-	-	-	[147]
*Nemopilema nomurai*	whole tissue	-	IC_50_ = 1.99 mg/mL	-	IC_50_ = 0.74 mg/mL	IC_50_ = 1.55 mg/mL	-	-	[161]
*Lobonema smithii*	whole tissue	-	8.13%	-	-	-	-	-	[174]
	oral arms	-	13.27%	-	-	-	-	-	
*Rhopilema hispidum*	umbrella	-	8.40%	-	-	-	-	-	[174]
	oral arms	-	10.026%	-	-	-	-	-	
*Lobonema smithii*	whole tissue	Fraction I (>10 kDa)	IC_50_ = 3.71 mg/mL	IC_50_ = 2.91 mg/mL	-	-	0.65 mmol FeSO_4_/g	-	[175]
		Fraction II (10–3 kDa)	IC_50_ = 0.85 mg/mL	IC_50_ = 1.15 mg/mL	-	-	0.27 mmol FeSO_4_/g	-	
		Fraction III (3–1 kDa)	IC_50_ = 0.95 mg/mL	IC_50_ = 0.91 mg/mL	-	-	0.24 mmol FeSO_4_/g	-	
		Fraction IV (<1 kDa)	IC_50_ = 1.11 mg/mL	IC_50_ = 0.89 mg/mL	-	-	0.28 mmol FeSO_4_/g	-	
*Tergillarca granosa*	whole tissue	*Met-Asp-Leu-Phe-Thr-Glu*	IC_50_ = 0.53 mg/mL	IC_50_ = 0.96 mg/mL	IC_50_ = 0.47 mg/mL	IC_50_ = 0.75 mg/mL	-	-	[176]
		*Trp-Pro-Pro-Asp*	IC_50_ = 0.36 mg/mL	IC_50_ = 0.54 mg/mL	IC_50_ = 0.38 mg/mL	IC_50_ = 0.46 mg/mL	-	-	

**Table 5:** Antioxidant activity for different marine species generated by different amino acid sequences evidenced by different specific enzymatic hydrolysis methods and different types of physicochemical analysis methods.

Fish Species	Source	Amino Acid Sequences/Amino Acid Fraction	Preparation Method	Antioxidant Activity	References
*Miichthys miiuy*	Swim bladder	Two chains (α1 and α2) as the major constituents with 115 kDa and 108 kDa	Enzymatic hydrolysis with pepsin	DPPH, ABTS, and hydroxyl radical- and superoxide anion radical-scavenging activity	[66]
*Miichthys miiu; Labeo rohita* *Tunuss albacares*; *Silurus triostegus*	Swim bladder	Collagen peptides	Enzymatic hydrolysis with pepsin	DPPH, ABTS	[86]
*Rhizostoma pulmo*	Whole body	Peptides with molecular weight < 3 kDa and between 3–10 kDa	Enzymatic hydrolysis with pepsin	DPPH	[147]
*Lophius litulo*	Skin	Amino acid with molecular weight range between 26–130 kDa	Enzymatic hydrolysis with pepsin	DPPH, ABTS, hydroxyl and superoxide anion radical-scavenging	[169]
*Rhizostoma pulmo*	Umbrella and oral arms	Peptide fractions with different ranges: <3 kDa; 3–10 kDa; 10–30 kDa; >30 kDa	Enzymatic hydrolysis with pepsin and collagenase	TEAC, ABTS	[177]
*Chanos Chanos*	Scales	Amino acid peptides with molecular weight < 3 kDa	Enzymatic hydrolysis with pepsin	DPPH, ABTS, lipide peroxidation inhibition, nitric oxide free radical scavenging	[178]
Fish (*Budu*)	Extract fish	Two novel peptides: LDDPVFIH and VAAGRTDAGVH,	Enzymatic hydrolysis pepsin	DPPH, ABTS superoxide anion radical scavenging	[179]
Small red scorpinfish *Scorpaena notata*	Whole body	*Leu-Val-Thr-Gly-Asp-Asp-Lys-Thr-Asn-Leu-Lys Asp-Thr-Gly-Ser-Asp-Lys-Lys-Gln-Leu*	Enzymatic hydrolysis with pepsin	DPPH	[180]
*Hypophthalmichthys molitrix*	Skin	Peptides with molecular weight < 1600 Da	Enzymatic hydrolysis with collagenase	DPPH, hydroxyl radical-scavenging activity	[181]
*Decapterus macarellus*	Skin	Collagen peptides	Enzymatic hydrolysis with collagenase	DPPH	[182]
Catfish	Skin	Amino acid peptides with 11–135 kDa	Pepsin, collagenase, and trypsin hydrolysis	DPPH, FRAP ability	[183]
*Caranx ignobilis*	Bone	Collagen peptides	Hydrolysis with collagenase enzyme	DPPH, FRAP ability	[184]
*Thunnus albacares*	Skin	Collagen peptides	Enzymatic hydrolysis with alkalase	DPPH, ABTS	[101]
*Tilapia*	Bone	Amino acids: *Glu*, *Lys Gly*, and *Pro*	Enzymatic hydrolysis with alkalase	DPPH, superoxide anion radical scavenging	[185]
*Cyprinus carpio*	Skin	Amino acid fractions: PF1 > 30 kDa; PF2 10–30 kDa; PF3 3–10 kDa and PF4 < 3 kDa	Enzymatic hydrolysis with alkalase	DPPH, hydroxyl radical-scavenging activity, FRAP ability	[186]
*Theragra chalcogramma*	Skin	Amino acid fractions: I < 3 kDa, II 3–10 kDa, III 10–30 kDa, and IV > 30 kDa	Enzymatic hydrolysis with alkalase	TEAC, FRAP ability, nitric oxide free radical scavenging, ORAC	[187]
*Cynoglossus arel*	Skin and scales	Collagen peptides	Enzymatic hydrolysis with alkalase	DPPH, metal-reducing power, metal-chelating activity	[188]
Salmon	Scales	Peptide with molecular weight between 219–347 Da	Enzymatic hydrolysis with alkalase	DPPH, ABTS, FRAP ability	[189]
*Rhopilema hispidum*	Whole body	Peptide fractions with molecular weight < 10 kDa, consisting mainly of Gly, Glu, and Arg	Enzymatic hydrolysis with papain	DPPH, metal ion-chelating assays	[174]
Sea cucumber *Actinopyga lecanora*	Stone fish	Ston fish crude protein	Enzymatic hydrolysis with papain	DPPH, ABTS, FRAP ability	[190]
Sturgeon fish	Head, skin	Amino acid fractions	Hydrolyzed with papain and bromelain	ABTS, hydroxyl radical-scavenging activity	[191]
*Lates calcarifer*	Skin	Amino acid peptide chain with aromatic and hydrophobic structures	Enzymatic hydrolysis with papain	Protection against H_2_O_2_ damage; nitric oxide free radical scavenging	[192]
*Hypophthalmichthys* *molitrix*	Fish waste	Amino acid with 1201.31–1874.01 Da	Enzymatic hydrolysis with papain	ABTS, hydroxyl radical-scavenging activity, lipide peroxidation inhibition	[193]
*Actinopyga lecanora*	Stone fish	Crude protein: *Gly*, *Glu*, *Asp*, and *Ala*; papain-digested proteolysate *Gly*, *Glu*, *Ala*, and *Asp*	Enzymatic hydrolysis with papain in digested proteolysate	DPPH• (IC_50_ = 0.49 mg/mL), ABTS• (IC_50_ = 0.36 mg/mL) and FRAP value (0.29 mM FeSO_4_)	[194]
*Nemopilema nomurai*	Whole body	Peptides with different molecular weights	Enzymatic hydrolysis with alcalase, protamex, flavourzyme enzymes, papain, pepsin, trypsin, and bromelain	DPPH, FRAP ability, hydroxyl and superoxide anion-scavenging activity	[161]
*Lobonema smithii*	Whole tissue	Amino acids—*Gly*, *Cys*, *Glx*, and *Asx*; peptide fraction	Enzymatic hydrolysis with alcalase, flavourzyme, and papain hydrolysis	DPPH, ABTS, FRAP ability	[174,175]
*Acaudina molpadioides*	Whole body	Amino acid from peptides with molecular weight < 1 kDa	Enzymatic hysrolysis with papain, pepsin, trypsin, and neutrase	DPPH, ABTS	[194]
*Katsuwonus pelamis*	Sales	Different peptide fractions: TGP5, TGP7, and TGP9	Enzymatic hydrolysis with papain, trypsin, and proteases—pepsin, neutrase, and alcalase	DPPH, hydroxyl and superoxide anion radical-scavenging activity	[195]
*Katsuwonus pelamis*	Skin	Amino acids: *Gly*, *Hyp*, *Pro*, and *Ala*	Enzymatic hydrolysis with trypsin, neutrase, papain, pepsin, and alcalase	DPPH	[196]
*Pseudosciaena polyactis*	Scales	Amino acid peptides with different chains: RCP1, RCP2, RCP3, RCP4, RCP5, and RCP6	Enzymatic hydrolysis with neutrase, pepsin, papain, trypsin, flavourzyme, and alcalase	DPPH, hydroxyl and superoxide anion radical-scavenging activity	[197]
Red lionfish (*Pterois volitans* L.)	Fish gutted, skinless fillets	The resulting peptide fractions exhibited high contents of amino acids	Enzymatic hydrolysis with trypsin, pepsin, chymotrypsin, and visceral enzymes	The highest copper-chelating activity, the highest iron-chelating activity, and β-carotene bleaching	[198]
*Miichthys miiuy*	Swim bladder	Collagen peptides	Enzimatic hydrolysis with pepsin, alcalase, neutrase, papain, and pepsin	DPPH, hydroxyl and superoxide anion radical scavenging	[199]
*Thunnus obesus*	Skin	Amino acids: Arg, Lys, Phe, and Tyr	Enzymatic hydrolysis with bromelain, papain, pepsin, and trypsin	DPPH, reducing power	[200]
*Mottle skate*	Cartilages	Amino acid peptide chains	Enzymatic hydrolysis with trypsin, chymotrypsin, and papain	DPPH, ABTS	[201]
Starry triggerfish *(Abalistes stellaris)*	Starry triggerfish muscle	Peptides derived from hydrolyzed fish protein	Enzimatic hydrolysis with trypsin	DPPH, ABTS, FRAP ability, and metal-chelating activity	[202]
*Nibea japonica*	Swim bladder	SNNH-1 (collagen peptide)—*Gly*, *Ala*, *Pro*, and *Hyp*	Enzymatic hydrolysis with neutrase	DPPH, ABTS, hydroxyl radical- and superoxide anion-scavenging activity	[203]
*Acaudina molpadioides*	Whole body	Amino acid from peptides with molecular weight < 1 kDa	Enzymatic hydrolysis with neutrase papain, pepsin, and trypsin	DPPH radical-scavenging activity, ABTS radical-scavenging activity	[194]
*Channa striata*	Scales	Collagen peptides	Enzymatic hydrolysis with protease	DPPH	[204]
*Chanos Chanos*	Skin	Collagen peptides	Enzymatic hydrolysis with protease	DPPH. ABTS	[205]
Salmon	Skin	Amino acid fractions: UF1 > 3 kDa and UF2 < 3 kDa	Digestion with protease from *Vibrio* sp.	DPPH, hydroxyl radical-scavenging activity, protection against H_2_O_2_ damage, (ORAC)	[206]
Sardine *(Sardina pilchardus)*	Head, scales, skin, blood,	SPH amino acid composition	Enzymatic hydrolysis with BSY protease; hydrolysis with 6 M HCl at 110 °C for 24 h.	FRAP ability	[207]
Fish *Conger myriaster*	Skin	Collagen peptides	The diethyl ether extracts of the skin	DPPH	[208]
Fish *Anguilla japonica*	Skin	Collagen peptides	The diethyl ether extracts of the skin	DPPH	[208]
*Oreochromis niloticus*	Skin	Amino acid fractions: I < 1 kDa, II 1–5 kDa, and III > 10 kDa	Extraction with crude enzyme solution from tuna stomach	ABTS, FRAP ability	[209]
Malaysian fish sausage *(Keropok Lekor)*	By-products	Native collagen and gelatine	Enzymatic by *Lactobacillus casei* fermentation	DPPH (82.8–88.4%) for fermented FBPs, DPPH (78.9%) for unfermented FBPs	[210]
*Johnius dussumieri*	Skin	Fractions with different molecular weights: >10 kDa, 5–10 kDa, 3–5 kDa, 1–3 kDa, and <1 kDa	Hydrolysis with visceral proteases extracted from the gastrointestinal (GI) tract of fish	DPPH, ABTS, FRAP ability, β-carotene bleaching prevention	[211]
Mackerel (*Scomber Japonicus*)	Fish	Ten peptides were synthesized	Enzymatic, sub-critical water hydrolysis, gamma irradiation, and chemical hydrolysis	DPPH (36.34%) and the highest SOD-like activity	[38]
Bigeye tuna	Skin	Peptides with low molecular weight < 600 Da	Subcritical water hydrolysis	DPPH, ABTS, FRAP ability and metal-chelating activity	[212]
Sea bream Sea bass	By-products, heads, bones	Amino acid profile of residues (gills, heads, and bones) from sea bass and sea bream	Collagen extraction with solvents extraction with pulsed electric fields	DPPH, ABTS, FRAP assay, and ORAC assay	[213]
*Sander lucioperca* and *Rutilus rutilus lacustris*	Fish lenses	Protein-free extracts from the fish lenses	The presence of ovothiol A (OSH) in the fish lenses of vertebrates	High concentrations of OSH level	[214]

**Table 6:** The biological activities of collagen hydrolysates, collagen peptides, and amino acid sequences from different marine organisms with results in the treatment of different diseases are systematized.

Marin Organism Type	Species	Organ/Protein/Peptide Fraction/Amino Acid Sequence	Biological Activity	Results in Treating Diseases	References
Fish	Barred mackerel	Skin fish gelatin/highest percentages of amino acid GPAQRID	Anticancer MCF-7 line cells Antioxidant (DPPH)	Nine fractions were obtained from the hydrolysis of fish skin gelatin. Fraction F1 was the most active fraction for antioxidant and cytotoxic activity against MCF-7-line cells	[217]
Fish (cold-water)	Rainbow trout	Skin—the collagen peptide fractions < 3 KDa	Anticancer HCT—116 cell line Antioxidant	The isolated peptide fractions from skin on HCT-116 cancer cells have cytotoxic properties. Skin hydrolysate showed antioxidant properties	[218]
Fish	Cod	Skin/two peptides: GEIGPSGGRGKP GKDGDAGPK and GFSGLDGAKGD	MMP are used for processes of angiogenesis and tumor metastasis	MMP inhibitory activity two peptides were found to exhibit a significant inhibition of MMP-1, p-ERK, and p-p38	[219]
Tunicate	*Eudistoma* cf. *rigida*	Lejimalides (A-D) unique 24-membered polyene macrolides	Antitumor	In vitro cytotoxic activity	[220]
*Mollusca bivalva*	*Arca subcrenata*	A polypeptide fraction with a MW of 20,491.0 Da	Antitumor activity in vitro and in vivo; antioxidant action	Purified polypeptides were treated for HeLa and for HT-29	[215] [221]
Mussel	*Mytilus edulis*	The 50 kDa fraction contains 56% of the proteins with important amino acids	Anti-proliferative agent	The 50 kDa fraction showed immortalized cancerous cell lines and mortality rate against BT549 cell lines, HCT15, A549, and PC3	[215] [219]
Fish	Tilapia fish *Oreochromis niloticus*	Skin/*Gly-Pro*-type peptides	Diabetes and DPP-IV-inhibitory activities	The DPP-IV-inhibitory activity of synthetic *Gly-Pro* was studied. It is used to treat people with type 2 diabetes.	[225]
Fish	*Hippoglossus stenolepis*	Skin/SPGSSGPQGFTG, GPVGPAGNPGANGLN, and PPGPTGPRGQPGNIGF	Diabetes	Displayed in vitro DPP-IV-inhibitory activity and were used for an in vivo antihyperglycemic experiment	[226]
Fish	*Oreochromis niloticus*	Skin/IPGDPGPPGPPGP, LPGERGRPGAPGP, and GPKDRGLPGPPGRDGM	Diabetes	Antihyperglycemic activity	[226]
Fish	Walleye pollock	Skin/collagen peptides	Anti-obesity activity	Attenuated obesity and modulated gut microbiota;	[227]
Jellyfish	*Diplulmaris antarctica*	Hydrolyzed collagen peptides	Anti-obesity activity	decrease the body weight by decreasing the level of fasting blood glucose	[228]
Fish—Atlantic Salmon	*Salmo salar*	Bone—small-molecular-weight peptide rich in *Gly-X-Y* structure	Osteoarthritis	Showed potential to be a novel and safe dietary supplement for helping anti-inflammatory activities, ultimately hindering osteoarthritis development	[229]
Fish	*Silver carp*	Skin—collagen hydrolysates OG or PG	Cardiovascular diseases antiplatelet peptides	The results showed that the antiplatelet peptides have increased antioxidant capacity beneficial in cardiovascular disease	[230]
Fish	*Salmo Salar*	Skin—multifunctional peptides FAGPPGGDGQPGAK and IAGPAGPRGPSGPA.	Atherosclerosis, antiplatelet aggregation and antioxidation activity	Atherosclerosis treatment protects against endothelial injury, platelet aggregation, and thrombosis. Aids anti-inflammation and endothelial damaging protection	[231]
Fish	*Saurida elongata*	Skin—RYRP	Hypertension	ACE-inhibitory activity with an IC_50_ value of 52 µM.	[185]
Fish	*Hemiramphus far*	Skin—collagen peptides	Hypertension	ACE-inhibitory activity 80.76% at 1 mg/mL	[232]
Fish	*Scorpaena notata*	By-product—PHSRSKGFPGP, GZKSVPQVR, and VQGKSPBV	Hypertension	ACE-inhibitory and antioxidant activity; IC_50_ values of 0.98, 1.69, and 1.44 μM	[233]
Fish *Nile Talpia*	*Oreochromis niloticus*	Skin—GIV, GAPGF, GFAGPA, SGDIGFPGPK, and GIPGPIGPPGRP MW less than 1.2 kDa	Moderate hypertension	The resulting hydrolysate had an ACE-inhibitory activity (IC_50_) of 1.2 mg/mL, which was slightly reduced by simulated gastrointestinal digestion.	[234]
Pearl oyster	*Pinctada fucata* *martensii*	HLHT and GWA	Hypertension	ACE inhibitory	[235]
Sea cucumber	*Stichopus japonicus*	Peptide fractions: EIYR, LF, and NAPHMR	Hypertension	ACE-inhibitory activity IC_50_ of 260.22 μM	[236]
Jellyfish	*Rhopilema esculentum* *kishinouye*	Peptide fraction: SY	Hypertension	ACE-inhibitory activity IC_50_ of 1164.179 μM	[237]
Fish	Skate *Raja kenojei*	Skin—ZGYRPLRGPQFL	Anti-Alzheimer’s, neuroprotective	Inhibitory peptides for β-secretase have been identified such as *Pro-Glu-Phe-Leu*.	[238] [240]
Cyprinidae Fish	*Ctenopharyngodon idella*	Skin—PYSFK—MW: 640.74 Da, GFGPZL—MW: 618.89 Da, VGGRPP—MW: 484.56 Da	Anti-Alzheimer’s, neuroprotective, antioxidant activity	Neuroprotection and antioxidant activity of these three peptide sequences	[238] [241]
Fish	*Oncorhynchus keta*	Skin—polypeptides	Anti-Alzheimer’s, neuroprotective	Anti-acetylcholinesterase, increasing hippocampus phosphorylation	[238] [242]
Seahorse	*Hippocampus trimaculatus*	Amino acids GTZDZLDK MW 906.4 Da	Neuroprotective	Neuroprotective effects against Ab42-induced neuronal death in PC12 cells	[215] [243]
Fish	*Tilapia*	Skin—collagen peptides	Oral mucosal ulcers	The effects of collagen peptides on the healing of oral mucosal ulcers in a rat model were macroscopically and microscopically analyzed in vivo	[244]
Fish	*Tilapia* scales	Collagen peptides	Oral and maxillofacial tissue regeneration	In vitro periodontal tissue regeneration	[245]
Fish	Sea bass *Lates calcarifer*	Hydrolyzed collagen from sea bass	Wound healing, antioxidant, and anti-obesity activities	HC and the HC-EGCGG conjugate showed anti-inflammatory activity by inhibiting the production of nitric oxide and tumor necrosis factor-α in lipopolysaccharide-induced RAW264.7 cells	[192]
Fish	Sea bass *Lates calcarifer*	Skin—antioxidant peptides (PO, POG)	Wound healing	Antioxidant peptides from Asian sea bass skin containing *Pro-Hyp* and *Pro-Hyp-Gly* increase wound healing by accelerating fibroblast mobility to injured tissue	[249]
Fish	*Nibea japonica*	Swim bladders—collagen peptides with GAPO	Wound-healing activity and antioxidant activity	Collagen peptides from collagen sponge produced accelerated wound healing. Mice treated with the collagen sponge had significantly reduced levels of interleukins	[92]
Fish	*Clarias batrachus*, *Pangasius pangasius*	Skin—peptide fractions in the range of 1–3 kDa molecular weight	Anti-inflammatory activity	The suppression of inflammatory proteins (TNF-α, IL6, NFκB, and p-IκB) by the peptide fractions confirmed the anti-inflammatory activity	[250]
Fish	*Thunnus obesus*	Collagen peptides from skin (TSCP) and from bones (TBCP) with <1 kDa	UV protector of skin, photoaging	Excellent anti-photoaging activity, improving cell viability and inhibiting skin water loss. Attenuated skin photoaging	[251]
Fish	Black pomfret (Parastromateus niger)	Peptides	Anti-aging effects	Natural bioactive peptides have been shown to improve the effects of skin aging due to free-radical-scavenging activity and anti-aging peptides	[252]
Fish	Hydrolyzed fish cartilage	Collagen peptides	Anti-aging activity	Measurements of skin wrinkles, dermis echogenicity and thickness, and morphological and structural characteristics of the skin were performed	[253]
Fish	*Salmo salar*	By-products—TPQVHIAVDKF	Anti-allergic activity	Subsequently, the novel eleven-amino-acid peptide, identified as TPEVHIAVDKF, was found to exert anti-allergic activity	[254]
Fish	By-product from fish scales	Fish scale peptides and other compounds like hydroxyapatite	Treating malnutrition	Each component has multiple beneficial properties for the human body as an antioxidant in the treatment of malnutrition	[255]
Fish	Pacific cod	Skin gelatin; GPAGPHGPPGKDGR, AGPHGPPGKDGR, and AGPAGPAGAR	Iron deficiency treatment	GPAGPHGPPGKDGR and AGPHGPPGKDGR supplied additional iron binding sites. This study suggests a potential application of gelatin-derived peptides as novel carriers to combat iron deficiency	[256]

## Abbreviations

ASC	Acid-soluble collagen
PSC	Pepsin-soluble collagen
WSG	Water-soluble gelatin
ROS	Reactive oxygen species
FTIR	Fourier-transform infrared spectroscopy
HPLC	High-performance liquid chromatography
DPPH	2,2-Diphenyl-1-picrylhydrazyl
ABTS	2,2′-Azinobis(3-ethylbenzothiazoline-6-sulfonic acid
FRAP	Ferric ion reducing antioxidant power
ACE	Angiotensin-converting enzyme
IL	Interleukin
MMP	Matrix metalloproteinase
